# IGLV3‐21^R110^ and ibrutinib treatment: Results from the double‐blind, randomized, placebo‐controlled GCLLSG CLL12 trial in early‐stage CLL

**DOI:** 10.1002/hem3.70385

**Published:** 2026-06-15

**Authors:** Deyan Y. Yosifov, Sandra Robrecht, Adam Giza, Palash C. Maity, Armin Riecke, Christof Schneider, Billy M. C. Jebaraj, Rashmi P. Dheenadayalan, Hassan Jumaa, Marc Young, Manish Kumar, Lothar Müller, Ursula Vehling‐Kaiser, Michael Eckart, Werner Freier, Björn Schöttker, Tobias Gaska, Marcel Reiser, Anna‐Maria Fink, Kirsten Fischer, Barbara Eichhorst, Michael Hallek, Petra Langerbeins, Eugen Tausch, Stephan Stilgenbauer

**Affiliations:** ^1^ Division of CLL, Department of Internal Medicine III Ulm University Hospital Ulm Germany; ^2^ German Cancer Research Center (DKFZ) Heidelberg Germany; ^3^ Department I of Internal Medicine, Center for Integrated Oncology Aachen Bonn Cologne Duesseldorf Faculty of Medicine and University Hospital Cologne Cologne Germany; ^4^ German CLL Study Group University of Cologne Cologne Germany; ^5^ Institute of Experimental Cancer Research Ulm University Medical Center Ulm Germany; ^6^ Internal Medicine, Division of Hematology German Military Hospital Ulm Ulm Germany; ^7^ Comprehensive Cancer Center Ulm (CCCU) Ulm University Hospital Ulm Germany; ^8^ Institute of Immunology Ulm University Hospital Ulm Germany; ^9^ Study Centrum Unter Ems Practice for Oncology and Hematology Leer Germany; ^10^ Outpatient Clinic Landshut Germany; ^11^ Onkologische Schwerpunktpraxis Erlangen Erlangen Germany; ^12^ Medicinum Hildesheim Germany; ^13^ Hämatologisch‐onkologische Schwerpunktpraxis Würzburg Würzburg Germany; ^14^ Brüderkrankenhaus St. Josef Paderborn Germany; ^15^ Praxis Internistischer Onkologie und Hämatologie Frechen Germany

## Abstract

IGLV3‐21^R110^ is a point mutation enabling autonomous B‐cell receptor (BCR) signaling in chronic lymphocytic leukemia (CLL). It has been associated with shorter time to first treatment and overall survival, but its effects have not been evaluated in phase 3 clinical trials or in patients treated with Bruton tyrosine kinase inhibitors. Here, we analyzed samples from the phase 3 CLL12 study that compared ibrutinib vs. placebo in untreated early‐stage CLL patients with intermediate to very high risk of disease progression, while patients with low risk of disease progression were allocated to watch‐and‐wait (w&w). Four detection methods (targeted next‐generation sequencing, Sanger sequencing, multiplex IGLV3‐21^R110^‐specific PCR, and flow cytometry) were compared, yielding highly consistent results. Overall, 34/515 (6.6%) patients had productive *IGLV3‐21* rearrangements: 5/152 (3.3%) in w&w, 15/181 (8.3%) in placebo, and 14/182 (7.7%) in the ibrutinib group. Of these, 25 were IGLV3‐21^R110^‐positive (3/5, 12/15, and 10/14). IGLV3‐21^R110^ was associated with shorter event‐free survival (EFS) across all arms: w&w (hazard ratio [HR] 17.03, 95% confidence interval [CI 95%]: 4.99–58.13, *P* < 0.001), placebo (HR 2.31, CI 95%: 1.16–4.6, P = 0.017), and ibrutinib (HR 2.99, CI 95%: 1.17–7.65, P = 0.023). Multivariable analysis identified IGLV3‐21^R110^ as an independent prognostic factor for shorter EFS (HR 3.18, CI 95%: 1.73–5.83, P < 0.001). Notably, IGLV3‐21^R110^ was associated with reduced clinical effectiveness of ibrutinib. Ca^2+^‐flux and cell viability assays using patient‐derived BCRs expressed in murine B‐cells confirmed reduced ibrutinib efficacy in IGLV3‐21^R110^ cases, especially regarding inhibition of antigen‐stimulated signaling. In summary, IGLV3‐21^R110^ is an independent prognostic factor for shorter EFS in early‐stage CLL and reduces ibrutinib effectiveness clinically and in vitro.

## Graphical abstract

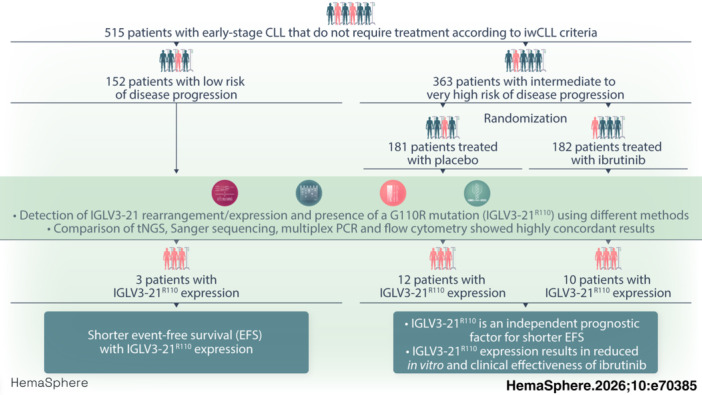

## INTRODUCTION

Chronic lymphocytic leukemia (CLL) is a B‐cell malignancy with a highly heterogeneous course, ranging from indolent disease that can be observed over years or even decades without requiring therapy to aggressive disease that necessitates immediate treatment after diagnosis.^1^ Signaling through the B‐cell receptor (BCR) is of key pathogenetic importance in CLL, contributing to CLL cell survival and proliferation.^2^ This is emphasized both by the high clinical effectiveness of drugs interfering with the BCR‐signaling pathway, such as inhibitors of the Bruton tyrosine kinase (BTK) or of phosphoinositol‐3‐kinases (PI3Ks), and the fact that variations in the immunologic characteristics of the BCR (hypermutation status of the immunoglobulin heavy‐chain genes [IGHV] and specific subsets of stereotyped BCRs) are among the most important pathogenic and prognostic biomarkers.^1^, ^2^, ^3^

Previous studies have established the existence of CLL BCRs capable of autonomous ligand‐independent signaling that arise as a result of mutations affecting specific amino acid (AA) residues in the variable regions of immunoglobulin light chains (LCs), thus creating conditions for homotypic interaction between neighboring BCRs.^3^, ^4^ Such receptors confer a survival advantage to the leukemic clone and might be an initial driver of CLL pathogenesis.^5^ The most prominent example involves the IGLV3‐21 LC, where a single point mutation of the last base of the joining (J) segment, leading to the substitution of a glycine with an arginine (G110R), can create an autonomously signaling BCR.^4^, ^6^ However, this is only possible if the mutation occurs in the allele *IGLV3‐21∗01* or *IGLV3‐21∗04,* as the other two known alleles (*02 and *03) do not fulfill the prerequisites for such a homotypic interaction (lysine at position 16 and tyrosine at position 49). IGLV3‐21 chains carrying the G110R substitution are denoted as IGLV3‐21^R110^ and are always present in subset #2 CLL cases, where they pair with IGHV3‐21, but they can form autonomous BCRs with other heavy chains (HCs) too.^4^, ^7^, ^8^, ^9^, ^10^ IGLV3‐21^R110^ has been detected in 3%–25% of CLL patients in different clinical cohorts.^7^, ^9^, ^11^, ^12^, ^13^, ^14^ CLL patients with IGLV3‐21^R110^ have shorter time to first treatment (TTFT) and shorter overall survival (OS), independently of their IGHV mutational status.^6^, ^9^, ^15^ A pooled analysis of clinical trials demonstrated that CLL patients with mutated IGHV (M‐CLL) belonging to subset #2 had shorter time to next treatment after chemo(immune)therapy‐based treatment compared to non‐subset #2 M‐CLL cases.^16^ However, analyses of clinical trial cohorts treated with venetoclax‐based therapy have not found evidence for differences in response rate, achievement of minimal residual disease, or progression‐free survival between patients with or without the IGLV3‐21^R110^ immunotype,^12^ and no data are available from clinical trials with BTK inhibitor‐based therapy. Here, we studied the potential role of IGLV3‐21^R110^ in the context of ibrutinib treatment in the German CLL Study Group (GCLLSG) CLL12 trial including early‐stage CLL patients, as well as in experimental model systems.

## MATERIALS AND METHODS

### Patient samples

The phase 3 CLL12 trial (https://www.clinicaltrials.gov/study/NCT02863718) enrolled previously untreated early‐stage CLL patients, who were either randomized to receive ibrutinib or placebo in case of intermediate to very high risk of disease progression or observed as a watch‐and‐wait group in case of low risk of disease progression. Inclusion criteria, as well as patient stratification and randomization procedures have been published previously.^17^, ^18^ Blood samples were obtained at enrollment of the patients at the participating clinical centers and shipped to the diagnostic laboratory at the University Hospital in Ulm. The patient consent included permission to use the samples for the current study. Peripheral blood mononuclear cells (PBMCs) were isolated from the blood samples by density gradient centrifugation in Leucosep® tubes (Greiner Bio One, Frickenhausen, Germany). A portion of each PBMC sample was viably frozen and stored at −196°C, and the rest was used for isolation of B‐cells by positive immunomagnetic selection with CD19 MicroBeads (cat. No. 130‐050‐301; Miltenyi Biotec), adhering to the manufacturer's protocol. The purity of the CD19^+^ fraction was ascertained by flow cytometry (median 95.7%, interquartile range 92.4%–97.3%). Cell pellets (CD19^+^) were stored at −80°C until the time of analysis.

### Isolation of nucleic acids

DNA and total RNA were isolated from CD19‐positive cells using the AllPrep kit (Qiagen) according to the manufacturer's protocol. DNA and RNA concentrations were measured using a Qubit® fluorometer (Thermo Fisher Scientific).

### Targeted next‐generation sequencing (tNGS)

All DNA samples were sequenced by tNGS using a custom panel covering genes relevant for CLL pathogenesis including *TP53, NOTCH1, SF3B1,* and *IGLV3‐21*, as well as the *IGLJ1‐IGLC1*, *IGLJ2‐IGLC2,* and *IGLJ3‐IGLC3* gene segments.^19^ Libraries were prepared according to the Illumina AmpliSeq protocol. Sequencing was performed on an Illumina MiSeq™ with the 600‐cycle MiSeq Reagent Kit v3 (Illumina Inc.). Alignment, variant calling, and annotation were performed with a custom bioinformatic pipeline relying on BWA‐MEM for alignment,^20^ VarScan2 for calling single‐nucleotide variants (SNVs) and short indels,^21^ ScanIndel^22^ for detection of medium‐sized indels and long deletions like the ones occurring during immunoglobulin gene rearrangement, IgCaller^23^ for additional analysis of immunoglobulin genes, and ANNOVAR^24^ for annotation of variants. Databases used for filtering of variants and estimation of pathogenicity included dbSNP (build 150), gnomAD genome and exome collections (v.2.1.1), COSMIC (v.98), and ClinVar (v.31.12.2022). The *IGLV3‐21* locus, eventual rearrangements affecting it, and mutations within the V and J regions were visually inspected using the Integrative Genomics Viewer (IGV) browser.^25^ Nucleotide sequences identified by IgCaller were additionally analyzed with IMGT/V‐QUEST^26^ to verify the identity of the *IGLV3‐21* allele and the *IGLJ* segment participating in the rearrangement.

### Sanger sequencing of immunoglobulin genes

The procedure for Sanger sequencing is described in the Supporting Information Materials and Methods (Supporting Information S1).

### Multiplex IGLV3‐21^R110^‐specific PCR (msPCR)

This method was performed analogous to the original publication, using the same primers − 2 forward primers binding to distinct regions of the *IGLV3‐21* gene, 2 R110‐specific reverse primers matching *IGLJ1* or *IGLJ2/3*, respectively, and a third pair of primers targeting *FBXW7* as an internal control.^9^

### Flow cytometric detection of IGLV3‐21^G110^ and IGLV3‐21^R110^

Immunophenotyping was performed using thawed PBMC samples as described previously.^6^ Please refer to the Supporting Information for further details.

### Expression of CLL‐derived BCRs and functional experiments

To analyze the BCR signaling capacity, patient‐derived immunoglobulin HC and LC variable regions were cloned and expressed in a murine pro‐B‐cell line derived from RAG2, λ5, and SLP65 triple‐knockout (TKO) mice, and expressing inducible ERT2‐SLP65 (EST) fusion protein.^6^, ^27^ As described previously, BCR expression in TKO‐EST cells enables analyses of both cell‐autonomous signaling upon activation of the EST fusion protein with 4‐hydroxytamoxifen and antigen‐ or cross‐linking‐induced signaling when anti‐κ/λ antibodies are also added (Southern Biotech). Briefly, TKO‐EST cells were cultured in IMDM medium (PAN‐Biotech) supplemented with 10% FBS Premium (PAN‐Biotech), 10 U/mL penicillin and 100 µg/mL streptomycin, 2 mM l‐alanyl‐L‐glutamine, 5 µM β‐mercaptoethanol (all from Invitrogen), 10 mM HEPES (Sigma‐Aldrich), and recombinant murine IL‐7 (5‐10 µg/mL; ImmunoTools) at 37°C in a humidified incubator with 7.5% CO_2_. Variable LC and HC domains from 8 CLL cases were cloned into a customized retroviral vector of pMIG‐w (Addgene ID 12282) origin, as a single open reading frame of p2a‐cleavable human λ‐LC and µ‐HC, followed by an internal ribosomal entry sequence linked to a green fluorescent protein reporter.^28^, ^29^, ^30^ Among the 8 receptors, 3 were from IGLV3‐21^G110^ cases and 5 were from IGLV3‐21^R110^ cases, including two previously characterized cases belonging to stereotypic subsets #2^31^ and #169,^8^ respectively. Additionally, we used a strongly reactive BCR from a CLL subset #201 case^30^ and a BCR derived from a healthy donor,^32^ respectively, as an experimental positive and negative control for cell‐autonomous signaling (Supporting Information S2: Table S1). Retroviral transduction was performed as described previously.^31^, ^33^ The Supporting Information contains a summary of this procedure, as well as of the protocols for Ca^2+^‐flux measurement, BH3 profiling, and the cell viability assay.

### Statistical analysis and data visualization

Genetic markers at baseline were compared using Fisher's exact test. TTFT (defined as the time from diagnosis to initiation of first treatment), event‐free survival (EFS; defined as the time from randomization/registration to active disease progression requiring treatment, initiation of subsequent treatment, or death), and OS (defined as the time from randomization/registration to death) were analyzed using Kaplan–Meier estimates and Cox proportional hazards regression modeling. Multivariable analyses were conducted using forward and backward selection procedures and considering baseline variables as candidates that were associated with EFS in univariable analyses. Statistical analyses were performed using SPSS v27 (IBM Corp.). All statistical tests were two‐sided, and P values were considered descriptive without adjustments for multiple testing.

GraphPad Prism® version 10.4.0 (GraphPad Software) and R were used for statistical analysis across different in vitro experiments. The used statistical tests are mentioned in the figure legends. Circos^34^ and the R package ComplexHeatmaps^35^ were used for additional visualizations.

P < 0.05 were considered statistically significant.

## RESULTS

### Comparison of methods for identification of IGLV3‐21^R110^

Samples were obtained from all 515 patients enrolled in the CLL12 study: 152 patients with low risk of disease progression in the watch‐and‐wait arm and 363 patients with intermediate to very high risk of disease progression who were randomized to placebo (*n* = 181) or ibrutinib (*n* = 182). Demographic and disease characteristics were balanced between the 2 treatment groups,^18^ whereas the observational group was characterized by a higher prevalence of cases with mutated IGHV and/or del(13q) as well as by a lower occurrence of del(17p), del(11q), trisomy 12, and mutations in known recurrently mutated genes (Supporting Information S1: Table S2).

All samples were sequenced by tNGS, and analysis of the results using IgCaller and ScanIndel revealed 33 samples with productive *IGLV3‐21* rearrangements and three samples with unproductive *IGLV3‐21* rearrangements due to out‐of‐frame junctions with resulting stop codons. Nine of the samples in the former group had the reference IGLV3‐21^G110^ sequence, 19 had IGLV3‐21^R110^, and the status of five samples, all of which used the *IGLJ1* segment, could not be determined because of suboptimal sequencing coverage (Supporting Information S2: Table S3). All 5 of these samples were identified as IGLV3‐21^R110^ using additional methods (see below).

All 36 samples with an *IGLV3‐21* rearrangement were additionally evaluated by Sanger sequencing of cDNA and by msPCR^9^ to evaluate the potential of tNGS for producing false‐positive results. Viably frozen cells were available from 20 of these patients and they were analyzed by flow cytometry (FC) too (Supporting Information S1: Figure S1). As several publications have shown that mutations in *SF3B1* and the usage of IGHV3‐21 HCs are significantly more common in IGLV3‐21^R110^ patients,^7^, ^9^, ^12^, ^14^ we reasoned that samples with *SF3B1* mutations or IGHV3‐21 usage and no rearrangement of IGLV3‐21 according to tNGS would be the most informative controls to analyze by the additional methods to assess whether tNGS produces false‐negative results. In total, 42 samples without the IGLV3‐21 rearrangement were analyzed by Sanger sequencing and msPCR, including 29 samples with *SF3B1* mutations and 9 with IGHV3‐21 usage. Twelve of these samples were additionally analyzed by FC (Supporting Information S1: Figure S1). Concordant results were obtained in 75 of all 78 patients tested by at least three methods (Supporting Information S2: Table S3). Among the three exceptions, one patient (#40627) had IGLV3‐21^R110^ according to both tNGS and msPCR, but was negative according to Sanger sequencing. Inspection of the tNGS results revealed a point mutation in *IGLV3‐21* in the binding site of the Sanger forward sequencing primer, which could explain the negative result by Sanger, and the sample was classified as IGLV3‐21^R110^. Another patient (#56057) had the IGLV3‐21*02/IGLV3‐21*03 genotype with usage of the IGLV3‐21*03 allele and no G110R mutation according to both tNGS and Sanger sequencing, but the normalized intensity of the 140 bp band in msPCR was above the threshold, thus formally fulfilling the criteria for IGLV3‐21^R110^ detection as specified by the authors of the method. However, in this method, only the forward primer for the longer 240 bp amplicon binds exclusively to the relevant IGLV3‐21*01 and IGLV3‐21*04 alleles, whereas the forward primer for the shorter 140 bp amplicon can bind to any *IGLV3‐21* allele. Thus, it was clear that the sample should be classified as IGLV3‐21^G110^. Regarding the third exception (patient #15100), tNGS could not provide conclusive evidence for an *IGLV3‐21* rearrangement and G110R mutation (detected in only 5 of 1424 reads) but Sanger sequencing, msPCR, and FC all classified the sample as IGLV3‐21^R110^, thus bringing the total number of IGLV3‐21^R110^‐positive patients to 25 and the total number of patients with productive *IGLV3‐21* rearrangements to 34.

### Prevalence of IGLV3‐21^R110^ and correlation with other genetic markers

**Figure 1:**
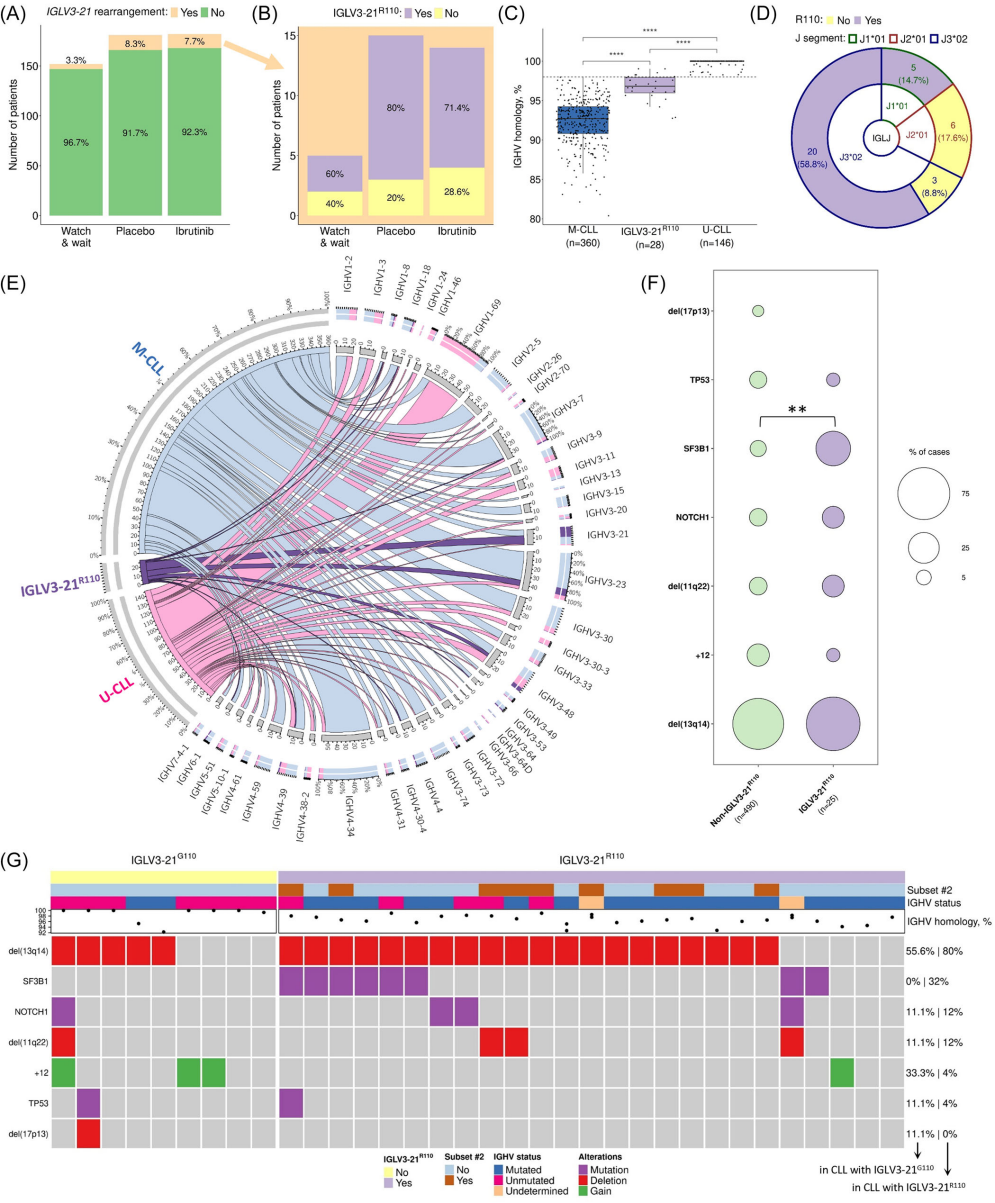
**Prevalence of IGLV3‐21** ^**R110**^ **in the CLL12 cohort and correlation with other genetic markers**. **(A)** Prevalence of the *IGLV3‐21* rearrangement in the three arms of the CLL12 trial. **(B)** Prevalence of the G110R mutation in the subgroup of patients with the *IGLV3‐21* rearrangement. **(C)** Combined box/scatter plots showing IGHV mutational load as the percentage of identity to the respective germline IGHV sequence. The dashed line depicts the threshold of 98% used for classifying CLL as M‐CLL or U‐CLL. Data from 534 IGHV‐defined CLL clones are shown, as some patients had more than one clone. ****P < 0.0001, Welch's ANOVA with Dunnett's T3 all‐pairs comparison post‐hoc test. **(D)** Used IGLJ segment and presence or absence of the G110R mutation among the cases expressing IGLV3‐21. **(E)** Circos plot showing IGHV genes used by CLL clones classified as either M‐CLL, U‐CLL, or IGLV3‐21^R110^ (*n* = 534, as in **(C)**). **(F)** Presence of specific chromosome aberrations and gene mutations in cases with and without IGLV3‐21^R110^. **P < 0.01, Fisher's exact test with Benjamini–Hochberg correction. **(G)** Oncoprint plot of the most common driver alterations in CLL cases with a productive *IGLV3‐21* rearrangement. Grouping is according to the absence or presence of the G110R mutation, and additional displayed parameters include belonging to subset #2 and IGHV mutational status. Patients with two CLL clones of diverging IGHV mutational status are classified as having undetermined overall status. Percentages represent the proportion of patients carrying each specific driver alteration among IGLV3‐21^G110^ cases (left) and IGLV3‐21^R110^ cases (right).

A productive *IGLV3‐21* rearrangement was present in 34/515 (6.6%) patients, including 5/152 (3.3%) low‐risk patients of the observational group, 15/181 (8.3%) patients of the placebo group, and 14/182 (7.7%) patients of the ibrutinib group (Figure 1A). The prevalence of the G110R mutation in patients with the *IGLV3‐21* rearrangement was 73.5% (25/34) in the total study population, and 60% (3/5), 80% (12/15), and 71.4% (10/14) per each group, respectively (Figure 1B). In line with published data,^6^, ^7^, ^9^, ^12^ IGLV3‐21^R110^‐positive patients had an intermediate IGHV mutational load, although 18 (72%) of them would be classified as M‐CLL based on the 98% sequence identity threshold (Figure 1C; Supporting Information S2: Table S4). The similarity of IGHV to the respective germline sequences ranged from 92.75% to 99.03%. The IGLV3‐21*04 allele was used by all 25 cases carrying the G110R mutation, as well as by 3 cases without the mutation (Supporting Information S1: Figure S2). Among the remaining 6 patients expressing IGLV3‐21 LCs, 4 used the IGLV3‐21*02 allele, and 2 used the IGLV3‐21*03 allele. All 5 cases that used the *IGLJ1* segment had the G110R mutation, whereas none of the 6 cases that used the *IGLJ2* segment had the mutation (Figure 1D). Nine of the 25 IGLV3‐21^R110^‐positive cases carried stereotyped IGH genes (36%), all of them belonging to subset #2 (19 major stereotyped subsets analyzed). Apart from IGHV3‐21, the other most commonly used HCs were IGHV3‐23 (7 cases) and IGHV3‐48 (4 cases; Figure 1E), which aligned with findings in other CLL cohorts.^7^, ^12^ None of the cases with IGHV3‐48 usage belonged to subset #169.

We used FISH and tNGS to characterize the genomic landscape of IGLV3‐21^R110^‐positive CLL in comparison to IGLV3‐21^R110^‐negative CLL. None of the IGLV3‐21^R110^‐positive cases had del(17p13), whereas mutations in *SF3B1* were significantly more common in IGLV3‐21^R110^‐positive cases than in IGLV3‐21^R110^‐negative cases (32% vs. 6.1%, P < 0.001, Figure 1F). None of the 9 IGLV3‐21^G110^ cases had mutations in SF3B1. Although three of the earlier studies reported mutual exclusivity of IGLV3‐21^R110^ and trisomy 12,^7^, ^9^, ^12^ we detected one case that had both (Figure 1F,G). The frequencies of del(13q14) and del(11q22) were not significantly different between IGLV3‐21^R110^‐positive cases and IGLV3‐21^R110^‐negative cases.

### Clinical implications of IGLV3‐21^R110^

**Figure 2:**
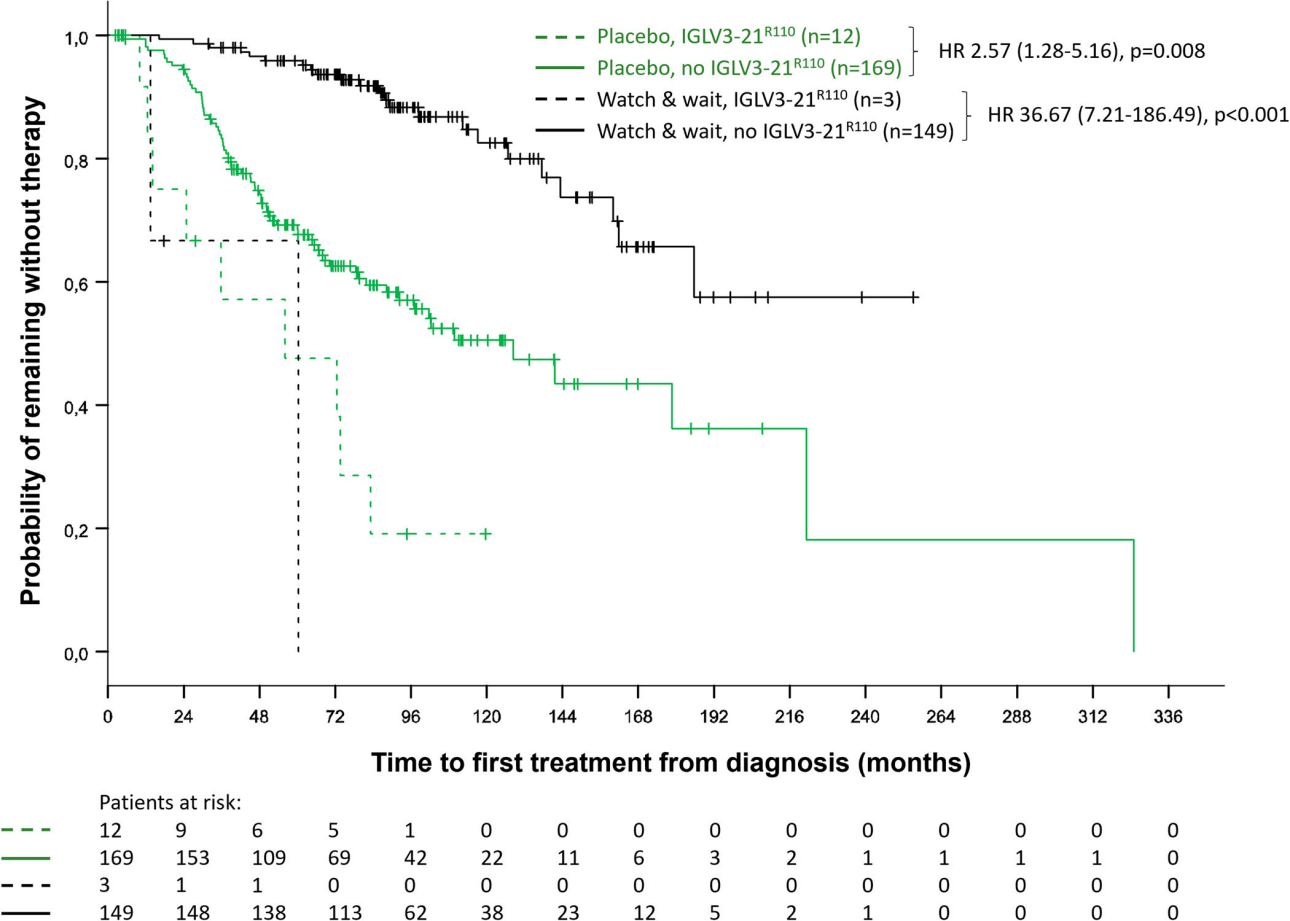
**Kaplan–Meier plot and risk table estimating time to first treatment in the watch & wait and placebo cohorts of the CLL12 trial.** The watch & wait group is depicted in black and the placebo cohort is shown in green. Within each of the two groups, patients expressing IGLV3‐21^R110^ (dashed lines) are compared against patients who do not express IGLV3‐21^R110^ (solid lines). Hazard ratios (HR) with 95% confidence intervals and P values were calculated using the Cox proportional hazards regression model with the Wald test.

First, we analyzed whether expression of IGLV3‐21^R110^ was associated with shorter TTFT. IGLV3‐21^R110^ positivity was associated with shorter TTFT both in the watch‐and‐wait arm (hazard ratio [HR] 36.67, 95% confidence interval [CI] 7.21–186.49, P < 0.001, albeit limited by very small sample sizes) and in the placebo arm (HR 2.57, 95% CI 1.28–5.16, P = 0.008; Figure 2). The ibrutinib arm was excluded from this analysis, as due to trial design, these patients received treatment early, before the criteria of the International Workshop on Chronic Lymphocytic Leukemia (iwCLL) were fulfilled.

**Figure 3:**
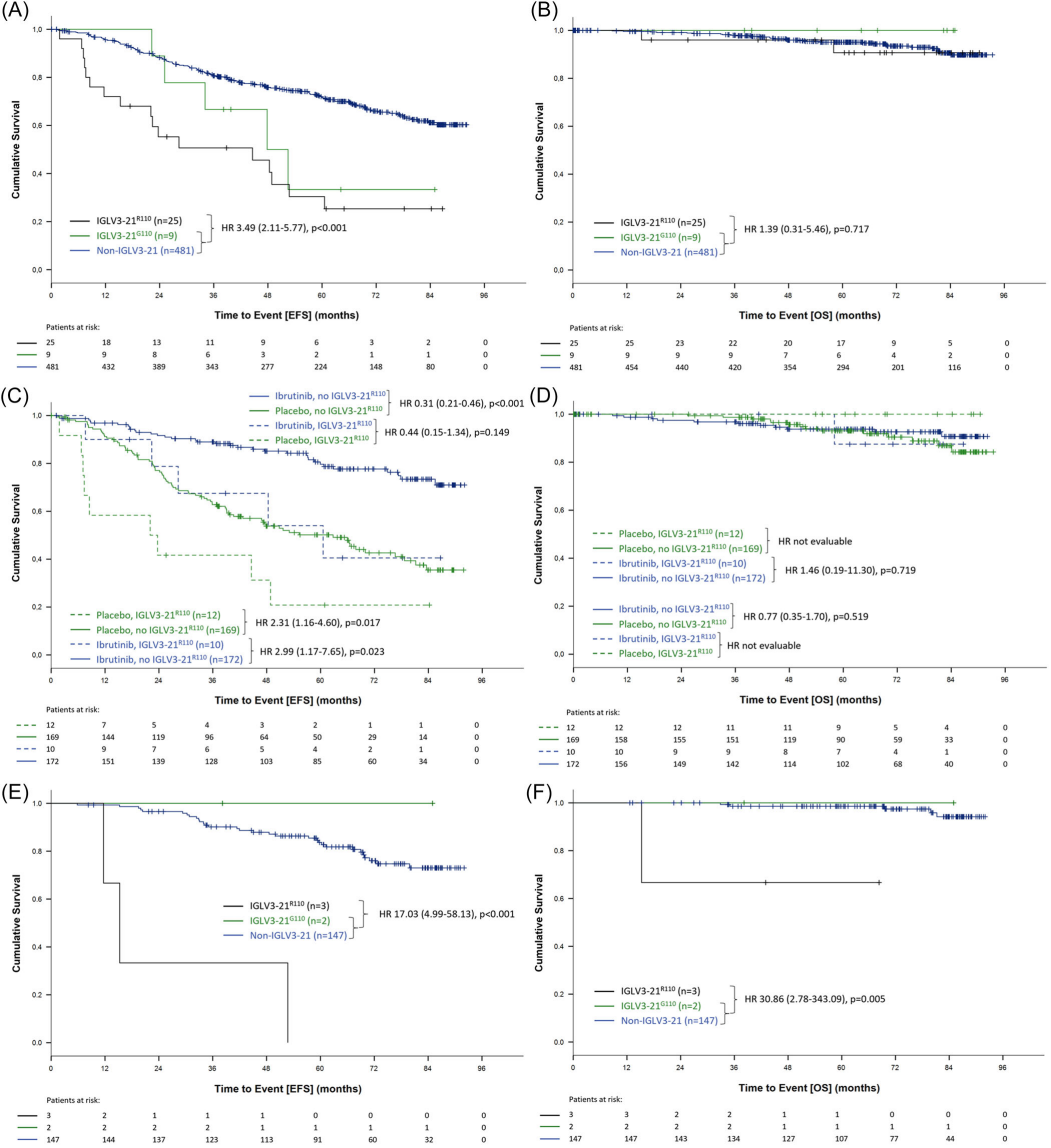
**Kaplan–Meier plots and risk tables estimating event‐free and overall survival in the CLL12 trial. (A**, **B)** EFS **(A)** and OS **(B)** of patients from all trial arms. The comparison is carried out between patients expressing IGLV3‐21^R110^ (black) and patients not expressing IGLV3‐21 light chains (blue) or expressing IGLV3‐21^G110^ (green), taken as one group. **(C**, **D)** EFS **(C)** and OS **(D)** of patients from the placebo (green) and ibrutinib (blue) arms. Within each treatment arm, patients expressing IGLV3‐21^R110^ (dashed lines) are compared against patients who do not express IGLV3‐21^R110^ (solid lines). **(E**, **F)** EFS **(E)** and OS **(F)** of patients from the observational arm. The comparison is carried out between patients expressing IGLV3‐21^R110^ (black) and patients not expressing IGLV3‐21 light chains (blue) or expressing IGLV3‐21^G110^ (green), taken as one group. Hazard ratios (HR) with 95% confidence intervals and P values were calculated using the Cox proportional hazards regression model with the Wald test. Statistics for all possible pairwise comparisons in **(A**, **B)** and **(E**, **F)** are provided in Supporting Information S1: Tables S5–S8.

Next, we evaluated the effects of IGLV3‐21^R110^ on the primary endpoint of the trial — EFS, as well as on OS. After a median follow‐up of 69.3 months, there were 166 events (32.2%) for EFS and 32 events for OS (6.2%). Considering the overall study population, IGLV3‐21^R110^‐positive CLL was associated with shorter EFS (HR 3.49, 95% CI 2.11–5.77, P < 0.001), but not OS (HR 1.39, 95% CI 0.31–5.46, P = 0.717) compared to non‐IGLV3‐21^R110^ cases (Figure 3A,B; Supporting Information S1: Tables S5‐S6). The presence of IGLV3‐21^R110^ was associated with shorter EFS both among patients with increased risk of progression in the treatment arms (Figure 3C) as well as in low‐risk patients of the watch‐and‐wait cohort (Figure 3E; Supporting Information S1: Table S7), or in all patients who were not treated with ibrutinib (Supporting Information S1: Figure S3). In the treatment cohort, the HRs were 2.31 (95% CI 1.16–4.6, P = 0.017) in the placebo group and 2.99 (95% CI 1.17–7.65, P = 0.023) in the ibrutinib group (Figure 3C). The estimated 5‐year EFS rates were 50.2% without versus 20.8% with IGLV3‐21^R110^ in the placebo group and 79.6% versus 54% in the ibrutinib group, respectively. Estimated 5‐year OS rates in the treatment arms of the trial were between 93.1% and 93.8% in the absence of IGLV3‐21^R110^, and its presence did not affect OS significantly (Figure 3D). The HR for shorter EFS with IGLV3‐21^R110^ was 17.03 (95% CI 4.99–58.13, P < 0.001) in the observational cohort of low‐risk patients (Figure 3E) and the HR for shorter OS was 30.86 (95% CI 2.78–343.09, *P* = 0.005; Figure 3F; Supporting Information S1: Table S8), but these results must be interpreted with caution, as the low‐risk cohort included only three patients with IGLV3‐21^R110^.

**Figure 4:**
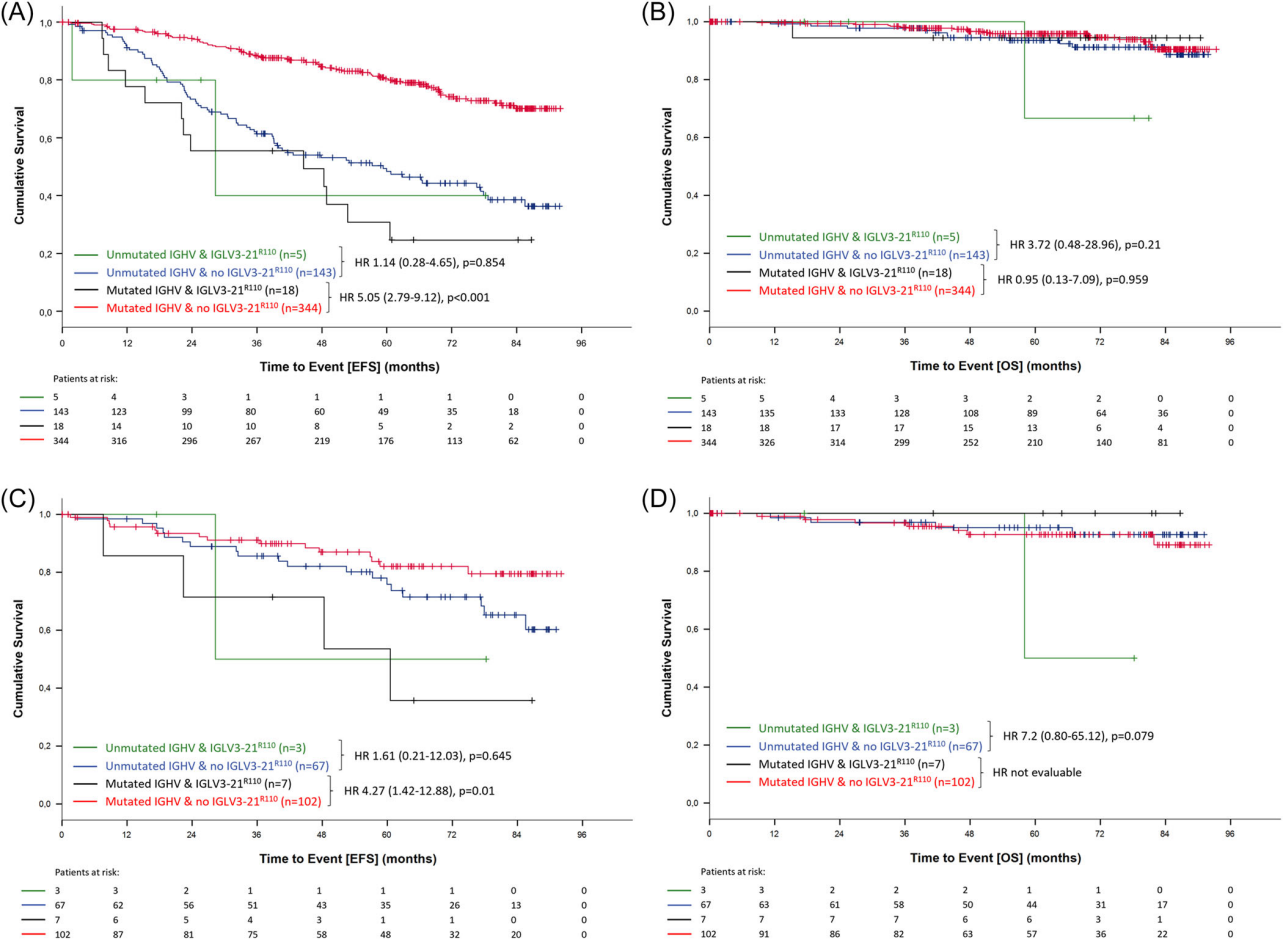
**Kaplan–Meier plots and risk tables estimating event‐free and overall survival in the CLL12 trial. (A**, **B)** EFS **(A)** and OS **(B)** of patients from all trial arms stratified according to their IGHV mutational status—unmutated (green and blue) or mutated (black and red). Within each of the two groups, patients expressing IGLV3‐21^R110^ (green or black, respectively) are compared against patients who do not express IGLV3‐21^R110^ (blue or red, respectively). **(C**, **D)** EFS **(C)** and OS **(D)** of patients from the ibrutinib arm separated according to their IGHV mutational status— unmutated (green and blue) or mutated (black and red). Within each of the two groups, patients expressing IGLV3‐21^R110^ (green or black, respectively) are compared against patients who do not express IGLV3‐21^R110^ (blue or red, respectively). Hazard ratios (HR) with 95% confidence intervals and P values were calculated using the Cox proportional hazards regression model with the Wald test. Statistics for all possible pairwise comparisons are provided in Supporting Information S1: Tables S9–S12.

In the full trial, IGLV3‐21^R110^‐positive M‐CLL patients had significantly shorter EFS than M‐CLL patients without the mutation (HR 5.05, 95% CI: 2.79–9.12, P < 0.001), while EFS was not significantly different between IGLV3‐21^R110^‐positive M‐CLL patients and U‐CLL patients regardless of their IGLV3‐21^R110^ status (HR 1.39, 95% CI 0.31–6.16, P = 0.666 for the comparison with IGLV3‐21^R110^‐positive U‐CLL patients; HR 1.58, 95% CI: 0.88–2.86, P = 0.126 for the comparison with IGLV3‐21^R110^‐negative U‐CLL patients; Figure 4A; Supporting Information S1: Table S9). These observations were also valid when the ibrutinib group was analyzed separately (Figure 4C; Supporting Information S1: Table S10). OS did not differ significantly between the groups defined by IGHV status and the presence of IGLV3‐21^R110^ (Figure 4B,D; Supporting Information S1: Tables S11‐S12).

When patients of the overall study population were stratified according to the presence or absence of *SF3B1* mutations, IGLV3‐21^R110^‐positive patients in any of the two resulting groups had shorter EFS than their respective IGLV3‐21^R110^‐negative counterparts (Supporting Information S1: Figure S4), which shows that the effects of IGLV3‐21^R110^ on EFS are not due to its association with *SF3B1* mutations. Similarly, the effect of IGLV3‐21^R110^ on EFS could not be explained by its association with subset #2, as among all IGLV3‐21^R110^‐positive patients, those that did not belong to subset #2 showed a trend of having even shorter EFS (Supporting Information S1: Figure S5).

Multivariable analysis was performed for the treatment cohort, including variables that were associated significantly with EFS in univariable analyses: IGHV status, IGLV3‐21 usage, IGLV3‐21^R110^ status, treatment arm (placebo or ibrutinib), serum β2‐microglobulin, del(17p), del(11q), and +(12) (Supporting Information S1: Table S13). IGLV3‐21^R110^ (HR 3.18, 95% CI 1.73–5.83, P < 0.001) was identified as an independent prognostic parameter along with treatment arm, IGHV status, and del(17p) (Table 1).

**Table 1:** Multivariable analysis of the effects of various prognostic variables on event‐free survival. The results of the univariable analysis are provided in Supporting Information S1: Table S13.

Prognostic factor	Hazard ratio (95% CI)	P value
Treatment with ibrutinib	0.251 (0.170–0.371)	<0.001
Unmutated IGHV	3.525 (2.449–5.076)	<0.001
del(17p)	3.357 (1.683–6.696)	<0.001
IGLV3‐21^R110^	3.178 (1.731–5.832)	<0.001

### Effects of IGLV3‐21^R110^ on the potency of ibrutinib as an inhibitor of BCR signaling

**Figure 5:**
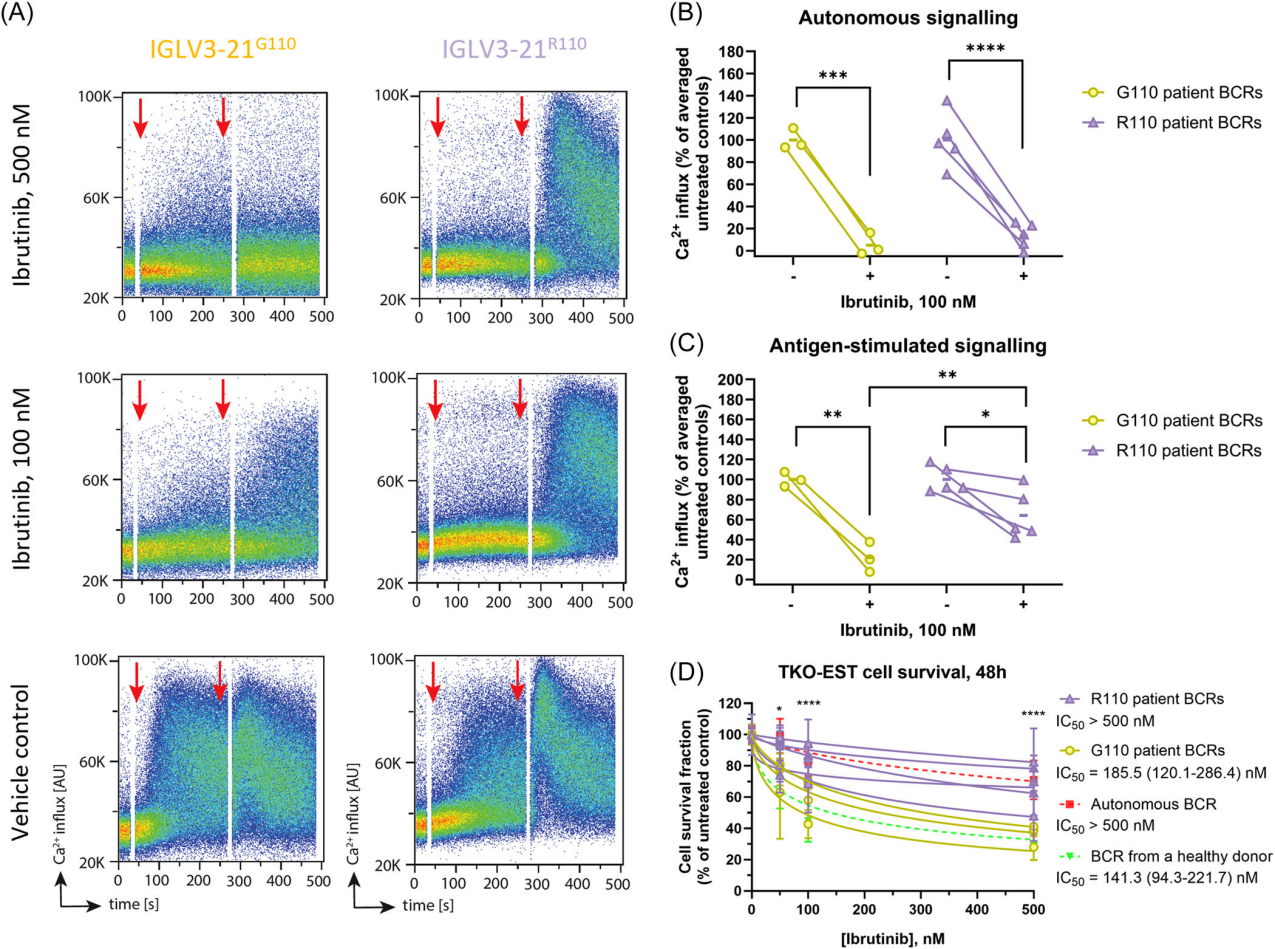
**IGLV3‐21** ^**R110**^ **reduces the effectiveness of ibrutinib. (A)** Ca^2+^ influx in TKO‐EST cells expressing a BCR from CLL patient 1 (IGLV3‐21^G110^, left panels) or from CLL patient 4 (IGLV3‐21^R110^, right panels). Cells were pre‐treated with vehicle or ibrutinib (100 or 500 nM, as indicated). On each density plot, the first arrow denotes the addition of 4‐hydroxytamoxifen (used to induce the ERT2‐SLP65 fusion protein and enable measurement of autonomous signaling) and the second arrow denotes the addition of anti‐κ/λ antibodies for measurement of antigen‐stimulated signaling. Additional data obtained with all used BCRs are provided in Supporting Information S1: Figures S7–S9. **(B**, **C)** Summarized data from the experiments described in **(A)** after normalization of the Ca^2+^ influx AUC to the mean of each respective untreated control. **(B)** Inhibition of autonomous signaling. **(C)** Inhibition of antigen‐stimulated signaling. Mean values are denoted by dashes. Statistical significance was evaluated using repeated‐measures two‐way ANOVA with uncorrected Fisher's LSD test. *P < 0.05; **P < 0.01; ***P < 0.001; and ****P < 0.0001. **(D)** Survival of TKO‐EST cells expressing BCRs from CLL patients with IGLV3‐21^G110^ or IGLV3‐21^R110^ light chains after treatment with ibrutinib for 48 h. Cell survival was measured using the CellTiter‐Glo® cell viability assay using a minimum of three replicates for each BCR. Each curve (four‐parameter logistic model) corresponds to a different patient BCR. An autonomously active BCR from a previously analyzed subset #201 CLL patient was used as a positive control. A BCR isolated from a naïve B‐cell from a healthy donor (IGHV4‐39/IGKV3‐15) was used as a negative control for autonomous signaling. Statistical evaluation was performed by 2‐way ANOVA and Šidák's multiple comparisons test, where the IGLV3‐21^G110^ and IGLV3‐21^R110^ groups were compared at each ibrutinib concentration level. *P < 0.05; ****P < 0.0001. Absolute IC_50_ values with 95% confidence intervals were determined from each curve and then geometrically averaged within each group.

As the above results showed that treatment with ibrutinib does not fully abrogate the negative effect of IGLV3‐21^R110^ on EFS, we hypothesized that ibrutinib cannot inhibit signaling through BCRs containing IGLV3‐21^R110^ with the effectiveness observed in the presence of other types of LCs, including IGLV3‐21^G110^. To test this hypothesis, we analyzed the signaling capacity (both cell‐autonomous and antigen‐dependent) of patient‐derived BCRs carrying either wild‐type IGLV3‐21^G110^ (*n* = 3) or mutated IGLV3‐21^R110^ (*n* = 5) and expressed on murine TKO‐EST cells, in the presence or absence of ibrutinib. All receptors (Supporting Information S2: Table S1) were expressed, assembled, and transported to the membrane surface as determined by FC (Supporting Information S1: Figure S6). Strikingly, BCRs with IGLV3‐21 LCs triggered both autonomously induced and anti‐λ crosslinking‐initiated Ca^2+^ influx, regardless of the presence of a G110R mutation (Figure 5A; Supporting Information S1: Figures S7 and S8). As expected, anti‐λ treatment resulted in stronger Ca^2+^ influx compared to autonomous signaling, as demonstrated by larger area under the curve (AUC).

Next, we analyzed the effect of ibrutinib on BCR signaling. Treatment with 100 nM ibrutinib was able to suppress both autonomous and antigen‐dependent signaling in all 3 tested IGLV3‐21^G110^ cases (Figure 5A–C and Supporting Information S1: Figure S9). In contrast, the response of cells expressing IGLV3‐21^R110^ was more nuanced — 4 of the 5 tested cases showed effective inhibition of autonomous signaling (Supporting Information S1: Figure S9A), while antigen‐dependent signaling was significantly decreased in only one case (Supporting Information S1: Figure S9B). Comparing the average AUC values per group, ibrutinib reduced autonomous signaling by 95% in IGLV3‐21^G110^‐expressing cells and by 86% in IGLV3‐21^R110^‐expressing cells (Figure 5B), whereas antigen‐stimulated signaling was reduced by 78% in IGLV3‐21^G110^‐expressing cells and by only 36% in IGLV3‐21^R110^‐expressing cells (Figure 5C). Thus, ibrutinib was significantly less effective as an inhibitor of antigen‐stimulated signaling in the presence of IGLV3‐21^R110^ (P = 0.006). Next, we compared the loss of viability of IGLV3‐21^G110^‐ and IGLV3‐21^R110^‐expressing cells when exposed to ibrutinib for 48 h. IGLV3‐21^R110^‐expressing cells showed significantly higher survival compared to IGLV3‐21^G110^‐expressing cells at all tested concentrations of ibrutinib and, moreover, their viability was similar to that of cells expressing an autonomously reactive BCR from a CLL subset #201 case used as a positive control (Figure 5D). The viability of IGLV3‐21^G110^‐expressing cells was lower and similar to that of cells expressing a BCR from a healthy donor.

Additionally, we performed BH3 profiling of primary CLL cells expressing IGLV3‐21^R110^ (*n* = 12) or not (*n* = 17). Both groups reacted with equally pronounced concentration‐dependent release of cytochrome c after treatment with either the activator peptide BIM or the MCL‐1 targeting sensitizer MS1, while IGLV3‐21^R110^‐positive cells were more sensitive to both BAD and venetoclax that act by inhibiting Bcl‐2 (Supporting Information S1: Figure S10A). Pretreatment of cells with 1 µM ibrutinib for 24 h significantly increased cytochrome *c* release in response to BAD and venetoclax in IGLV3‐21^R110^‐negative cells, but not in IGLV3‐21^R110^‐positive cells (Supporting Information S1: Figure S10B). Collectively, these results point to increased dependence of IGLV3‐21^R110^‐expressing CLL cells on Bcl‐2 and reduced additional priming in response to ibrutinib.

## DISCUSSION

In this early‐stage CLL cohort from the CLL12 trial, we observed a productive rearrangement of *IGLV3‐21* in 34 (6.6%) of 515 patients, and 25 of them (4.9% in total) were IGLV3‐21^R110^‐positive. The percentage of IGLV3‐21^R110^‐positive CLL cases in various studies has ranged from 3.5%–7.7% in cohorts collected by single institutions,^9^ probably on an all‐comer basis, to 25% in treatment trials like HOVON‐139 and HOVON‐141.^12^ It has been hypothesized that this discrepancy can be explained by enrichment of IGLV3‐21^R110^ cases in populations requiring therapy, as IGLV3‐21^R110^ CLL is associated with shorter TTFT.^6^ Geographical differences in the frequency of the IGLV3‐21*01 and IGLV3‐21*04 alleles might also play a role.^6^ The CLL12 trial has a unique study population including only patients without the need for treatment per iwCLL criteria.^36^ Thus, our study cohort was most likely not enriched in IGLV3‐21^R110^ cases and their observed percentage should be representative of the prevalence of IGLV3‐21^R110^ in the broad CLL patient population at diagnosis. The lower percentage of IGLV3‐21^R110^ cases in the low‐risk group (2%; 3/152) compared to the treatment group (6.1%; 22/363) hinted at an association of IGLV3‐21^R110^ with the risk factors (age >60 years, male sex, β2‐microglobulin 1.7–3.5 mg/L or >3.5 mg/L, ECOG performance status >0, thymidine kinase >10 U/L, unmutated IGHV status, del(11q), and del(17p)) that make up the GCLLSG score^37^ used for estimating the risk of disease progression and, respectively, for the allocation of patients to the observational or the treatment group. Indeed, an additional analysis showed the correlation of IGLV3‐21^R110^ with both serum thymidine kinase (P = 0.023) and β2‐microglobulin levels (P = 0.049; Supporting Information S1: Table S14).

Interestingly, all IGLV3‐21^R110^ cases in our cohort used either the IGLJ1 or IGLJ3 segment, and none used the IGLJ2 segment. Furthermore, all cases with IGLJ3 usage expressed exclusively the IGLJ3*02 allele and not the IGLJ3*01 allele, which has the same sequence as *IGLJ2*. Two other studies that have determined the usage of IGLJ segments report a similar picture with 0/19,^14^ resp. 0/24^15^ IGLV3‐21^R110^‐expressing CLL clones using IGLJ2, although the possible reasons were not elaborated. The reference AA sequence of IGLJ3*02 differs from IGLJ3*01 and resp. from IGLJ2 by a single residue, namely, IGLJ3*02 has a tryptophan (W) at position 98, while IGLJ3*01 and IGLJ2 have a valine (V) at the same position. IGLJ1 has a tyrosine (Y) at position 98 but also additional differences compared to IGLJ3*02. In light of the combined evidence, we hypothesize that a bulky AA residue at position 98 whose side chain is capable of hydrogen bonding is essential for the homotypic BCR–BCR interaction that is characteristic of IGLV3‐21^R110^‐positive CLL. Similar to other published data,^7^, ^12^, ^15^ we observed skewed usage of HCs in IGLV3‐21^R110^ cases, with a limited repertoire dominated by IGHV3‐21, IGHV3‐23, and IGHV3‐48, reflecting the indispensability of specific critical AA residues for homotypic BCR–BCR interactions in the context of IGLV3‐21^R110^.^3^, ^8^ Most IGLV3‐21^R110^ cases in CLL12 had borderline IGHV mutational status and none had 100% identity with the respective germline sequence, again corroborating earlier reports.^6^, ^7^, ^12^, ^15^

Previous retrospective analyses have suggested that IGLV3‐21^R110^ is a prognostic marker for shorter TTFT.^6^, ^7^, ^9^ Our data confirm these observations in a prospective trial including also patients who are classified as low risk according to standard criteria (GCLLSG score). Moreover, we show for the first time in a relatively large randomized phase 3 clinical trial that IGLV3‐21^R110^‐positive CLL patients have shorter EFS, and multivariable analysis identified IGLV3‐21^R110^ as an independent prognostic factor. Notably, while early treatment with ibrutinib significantly delayed progression to symptomatic disease, it could not fully abrogate the reduced EFS associated with the presence of IGLV3‐21^R110^. In line with this, our in vitro experiments with murine B‐cells expressing CLL‐derived BCRs demonstrated reduced ability of ibrutinib to inhibit antigen‐triggered signaling through the BCR/BTK pathway in the setting of IGLV3‐21^R110^ expression. Accordingly, these cells survived higher concentrations of ibrutinib. Interestingly, autonomous signaling through the BCR/BTK pathway was fully impaired upon ibrutinib treatment irrespective of IGLV3‐21^R110^ expression. Notably, induction of autonomous signaling in these experiments was not restricted to IGLV3‐21^R110^‐containing BCRs, which challenges the originally proposed model based on stereotypic CLL subset #2.^4^ Indeed, the role of specific LC–HC pairings has been increasingly recognized as an additional layer to the original classification of HC‐based major stereotypic CLL subsets and has been linked to distinct biological and clinical implications.^38^ Owing to the complex secondary and tertiary structures of BCRs, single‐point mutations affecting the HC can result in gain or loss of autonomous signaling, irrespective of IGLV3‐21^R110^ usage.^8^, ^39^ In other words, there could be IGLV3‐21^R110^‐containing BCRs that are not autonomously active, as well as IGLV3‐21^G110^‐containing BCRs that are autonomously active, as shown in the current study. Together, these observations raise the question of whether the CLL‐driving properties of IGLV3‐21^R110^ are really due to autonomous signaling or rather due to interaction with some as yet unidentified autoantigen(s). Our current results support this alternative hypothesis, as antigen‐dependent signaling through IGLV3‐21^R110^‐containing BCRs was poorly inhibited by ibrutinib compared to IGLV3‐21^G110^ cases. However, more observations will be necessary for a firm conclusion, preferably using model systems other than the TKO‐EST system used here to overcome the difficulties with studying BCR signaling in primary cells.

It must be considered that treatment with ibrutinib in the CLL12 trial was started early, in patients who would not normally be treated yet according to iwCLL guidelines. The prognostic and possibly predictive value of IGLV3‐21^R110^ for the effectiveness of ibrutinib and other state‐of‐the‐art therapies should be further studied in randomized trials including patients fulfilling the treatment indication criteria of the iwCLL. In light of our finding that IGLV3‐21^R110^‐expressing CLL cells have increased dependence on Bcl‐2 and, respectively, susceptibility to venetoclax, it might be expected that venetoclax would be more effective in IGLV3‐21^R110^‐positive CLL. It is well known that ibrutinib increases Bcl‐2 dependence of CLL cells and enhances sensitivity to venetoclax;^40^, ^41^ however, our dynamic BH3‐profiling results suggest that this synergistic effect might be less pronounced in IGLV3‐21^R110^‐positive CLL. For reference, the effect of IGLV3‐21^R110^ has already been evaluated in CLL patients undergoing first‐line treatment with obinutuzumab and venetoclax in the HOVON‐139 phase 2 trial, as well as in relapsed/refractory CLL patients treated with ibrutinib and venetoclax in the Dutch sub‐cohort of the HOVON‐141/VISION phase 2 trial, with the result that no evidence for a prognostic impact on the efficacy of targeted therapies could be found.^12^ However, the number of IGLV3‐21^R110^ patients in these trials was small, limiting the conclusions, and larger studies would be needed to establish optimal treatment strategies for IGLV3‐21^R110^‐positive CLL.

Our results might, at first glance, seem contradictory with the results of a recently published large French retrospective study by Bussot et al., who claimed longer progression‐free survival for subset #2 patients treated with BTK inhibitors and tried to extrapolate this finding to the broader group of all IGLV3‐21^R110^‐positive CLL patients, although they did not test for IGLV3‐21^R110^ in their study.^42^ However, in our study, only 36% of all IGLV3‐21^R110^‐positive CLL patients belonged to subset #2 and, moreover, the shorter EFS in the IGLV3‐21^R110^‐positive group was mostly driven by the non‐subset #2 cases—an important group of patients who remained ignored by the study of Bussot et al., also because they restricted their analysis only to patients expressing IGHV3‐21.^42^

Finally, our comparison of IGLV3‐21^R110^ detection methods showed very good concordance and demonstrated the feasibility of using a single tNGS panel for simultaneous detection of IGLV3‐21^R110^ and mutations in other recurrently mutated genes in CLL. Our results also show that caution is needed with any of the primer‐based approaches, as point mutations, which are common in the variable regions of immunoglobulins, can affect primer binding and can lead to false‐negative results, thus advocating for the parallel use of independent sets of primers covering the same region or orthogonal methods like FC to ensure higher reliability. Although we did not see any false‐negative results with FC, it has to be considered that AA residue changes in the vicinity of position 110 of the IGLV3‐21 LC can theoretically affect antibody binding and the reliability of this method.

In summary, IGLV3‐21^R110^ was identified as an adverse marker of independent prognostic impact for shorter EFS in early‐stage CLL with intermediate to very high risk of disease progression and was associated with reduced effectiveness of ibrutinib, both clinically and in vitro. It remains unclear to what extent these effects are mediated by autonomous signaling or by interaction of IGLV3‐21^R110^‐containing BCRs with a particular antigen. Future studies should resolve this question and assess the impact of IGLV3‐21^R110^ on the effectiveness of BTK inhibitors in closer‐to‐real‐life settings, that is, in patients fulfilling the treatment indication criteria of the iwCLL.

## AUTHOR CONTRIBUTIONS

**Deyan Y. Yosifov**: Conceptualization; methodology; investigation; data curation; formal analysis; project administration; software; validation; visualization; writing—original draft preparation; writing—review and editing. **Sandra Robrecht**: Formal analysis; methodology; validation; visualization; writing—review and editing. **Adam Giza**: Formal analysis; visualization. **Palash C. Maity**: Methodology; investigation; validation; formal analysis; visualization; writing—review and editing. **Armin Riecke**: Resources. **Christoph Schneider**: Resources; formal analysis; writing—review and editing. **Billy M. C. Jebaraj**: Conceptualization; methodology; validation; writing—review and editing. **Rashmi P. Dheenadayalan:** Methodology; investigation. **Hassan Jumaa**: Investigation; validation. **Marc Young**: Investigation; validation. **Manish Kumar**: Investigation; formal analysis. **Lothar Müller**: Resources. **Ursula Vehling‐Kaiser**: Resources. **Michael Eckart**: Resources. **Werner Freier**: Resources. **Björn Schöttker**: Resources. **Tobias Gaska**: Resources. **Marcel Reiser**: Resources. **Anna‐Maria Fink**: Resources. **Kirsten Fischer**: Resources; writing—review and editing. **Barbara Eichhorst**: Resources; writing—review and editing. **Michael Hallek**: Resources; writing—review and editing. **Petra Langerbeins**: Conceptualization; resources; writing—review and editing. **Eugen Tausch**: Conceptualization; resources; formal analysis; validation; supervision; writing—review and editing; funding acquisition. **Stephan Stilgenbauer**: Conceptualization; resources; formal analysis; validation; supervision; writing—review and editing; funding acquisition.

## CONFLICT OF INTEREST STATEMENT

S.R. reports personal fees from MSD outside the submitted work. A.R. reports personal fees outside the submitted work from AstraZeneca, BMS, Roche, and Regeneron. C.S. has received honoraria from AbbVie, AstraZeneca, Janssen‐Cilag, Lilly, MSD, and Novartis. A.‐M.F. reports personal fees from Janssen outside the submitted work. K.F. reports honoraria from Roche and AbbVie during the conduct of the study. B.E. reports grants and personal fees from Janssen‐Cilag, Roche, AbbVie, and Gilead, and personal fees from Novartis, Celgene, ArQule, AstraZeneca, Oxford Biomedica (UK), and grants from BeiGene outside the submitted work. M.H. received institutional research support from AbbVie, F. Hoffman‐La Roche, Gilead, Janssen‐Cilag, and Mundipharma during the conduct of the study. P.L. reports grants and other support from Janssen‐Cilag, BeOne, AstraZeneca, AbbVie, and F. Hoffman‐La Roche during the conduct of the study. E.T. reports grants and personal fees from Roche during the conduct of the study; grants, personal fees, and nonfinancial support from AbbVie; and personal fees and nonfinancial support from Janssen outside the submitted work. S.S. reports grants, personal fees, and nonfinancial support from AbbVie, AstraZeneca, Celgene, Gilead, F. Hoffmann‐La Roche, Janssen (Johnson & Johnson), and Novartis during the conduct of the study; and grants, personal fees, and nonfinancial support from AbbVie, AstraZeneca, Celgene, F. Hoffmann‐La Roche, Janssen, and Novartis outside the submitted work. The remaining authors declare no competing financial interests.

## ETHICS STATEMENT

This study was conducted in accordance with the Declaration of Helsinki and was approved by the Institutional Review Board of the University Hospital in Ulm, Germany.

## FUNDING

P.C.M. is supported by grants from the Fritz Thyssen Foundation (10.23.1.012MN) and the German Research Foundation (DFG CRC1279, Project B01 to Christian Buske). Open Access funding enabled and organized by Projekt DEAL.

## Supporting information

IGLV3‐21 CLL12 Supporting Information S1.

IGLV3‐21 CLL12 Supporting Information S2.

## Data Availability

The data that support the findings of this study are available on request from the corresponding author. The data are not publicly available due to privacy or ethical restrictions.
