# Supplementary material for: IGLV3‐21R110 and ibrutinib treatment: Results from the double‐blind, randomized, placebo‐controlled GCLLSG CLL12 trial in early‐stage CLL
Source: Hemasphere. 2026 Jun 15;10(6):e70385. doi: 10.1002/hem3.70385 (PMC13266578; doi:10.1002/hem3.70385)
Supplement: Supplementary file 1 — IGLV3‐21 CLL12 Supporting Information S1. [file HEM3-10-e70385-s002.pdf]

Supporting Information

IGLV3-21^{R110} and ibrutinib treatment: results from the double-blind, randomized, placebo-controlled GCLLSG CLL12 trial in early-stage CLL

Supplemental Materials and Methods 2
Supplemental Tables 4
Supplemental Figures 16
Supplemental References 29

Supplemental Materials and Methods

Sanger sequencing of immunoglobulin genes

IGHV genes were amplified by PCR from genomic DNA using primers binding to the leader/FR1 and IGHJ regions, following a previously described protocol.[1] IGLV3-21 was amplified from cDNA after reverse transcription of RNA with the LunaScript® RT SuperMix Kit (New England Biolabs, USA). The PCR was performed with primers binding to the FR1 region (5'-TCCTATGTGCTGACTCAGCCACC-3') and the constant region (5'-TGAAGATTCTGTAGGGGCCACTGTCTT-3').[2] All primers were synthesized and delivered by Eurofins Scientific, Luxembourg. Sequencing was performed using the BigDye® Terminator v3.1 Cycle Sequencing Kit (ThermoFisher Scientific, USA) and the unincorporated dye terminators were removed using the DyeEx 2.0 Spin Kit (Qiagen, Hilden, Germany). Capillary electrophoresis was performed on an Applied Biosystems® 3500 Genetic Analyzer and the data were analyzed using the Sequencing Analysis software v7.0 (Applied Biosystems, Foster City, USA). Stereotyped subsets were determined with ARResT/AssignSubsets (v.07.01.22)[3] and subset #2 was also confirmed with IMGT/V-QUEST (v.3.6.3).[4]

Flow cytometric detection of IGLV3-21^{G110} and IGLV3-21^{R110}

Immunophenotyping was performed using thawed PBMC samples as described previously.[5] Briefly, 5×10^6 cells were incubated on ice for 30 min in 50 µl of PBS with 3% fetal bovine serum (FBS) and the following antibodies: Brilliant Violet 510 (BV510)-conjugated anti CD19 (dilution 1:100; clone HIB19, cat. No. 302242, Biolegend), phycoerythrin-Cy7 (PE-Cy7)-conjugated anti-CD5 (dilution 1:100; clone L17F12, cat. No. 364008, Biolegend), Alexa Fluor 488 (AF488)-conjugated anti-IGLV3-21 (0.06 µg/ml; clone 42-1-D1-19[5]), Alexa Fluor 647 (AF647-conjugated anti-IGLV3-21^{R110} (0.06 µg/ml; clone 16-B2-18[5]) and unconjugated Fc blocker (dilution 1:100; cat. No. 564219, BD Bioscience). Afterwards, samples were washed twice, stained with SYTOX™ Blue Dead Cell Stain (dilution 1:10000; cat. No. S34857, ThermoFisher Scientific) and immediately measured. The results were analyzed in FlowJo 10.7.1 (TreeStar Inc., USA) by first gating on live cells, then on $CD5^+$/$CD19^+$ cells, and finally assessing IGLV3-21^{G110}/IGLV3-21^{R110} expression.

Retroviral transduction

In brief, using the GeneJuice® transfection reagent (Millipore), Phoenix-ECO cells were transfected with BCR-encoding retroviral plasmids along with the ecotropic packaging helper plasmid pCL-Eco (Addgene ID 12371). Mature retroviral particles were collected from the culture supernatant after 72 hours and used to transduce TKO-EST cells by the spin-infection method in the presence of 5 µg/ml polybrene (Millipore). BCR expression was assessed flow cytometrically 4-5 days after retroviral transduction by the percentage of GFP-positive cells and surface staining of membrane bound IgM BCR molecules using biotinylated anti-λ or -κ LC antibodies (Southern Biotech), streptavidin PE-Cy7 (Invitrogen) and an Alexa Fluor® 647-conjugated anti-IgM (Fc5µ) antibody (Jackson ImmunoResearch, Suffolk, UK).

Calcium flux measurement

For the cytosolic Ca^{2+} influx analyses, 1-2x10^6 transduced TKO-EST cells were loaded for 45 minutes at 37°C with 0.05 % Pluronic™ F-127 and 5 µg/ml of the Ca^{2+} indicator dye Indo-1 AM (both from Invitrogen) in IMDM supplemented with 2% FBS. Cells were washed and treated with desired

concentrations of ibrutinib or vehicle for 5 minutes at 37° C prior to analyses. Baseline signal for calcium was measured for 30 s followed by addition of 4 µM 4-hydroxytamoxifen to activate ERT2-SLP65 for assessment of cell-autonomous signaling. BCR cross-linking was achieved by subsequent treatment with 5 µg/ml goat anti-human κ/λ antibodies (Southern Biotech) to check antigen-induced BCR signaling. Live cell-based assays were evaluated on a BD LSRFortessa™ Cell Analyzer (BD Biosciences) with a UV laser and temperature-controlled tube holder. Data were plotted, quantified by area under the curve (AUC) above the baseline for each stimulation response within the experiment and analyzed as described before.[6]

Cell viability assay

BCR-expressing transduced TKO-EST cells were stained with CellTrace™ Far Red cell proliferation dye (Life Technologies) at a final concentration of 0.5 µM, according to manufacturer's instructions. $5x10^5$ stained cells were resuspended in IMDM containing 2% FBS and seeded in a 96-well flat bottom plate (200 µl/well), with or without presence of ibrutinib, and incubated at 37°C in a humidified incubator with 7.5% CO_2 for 48 hours. Thereafter, cells were stained with Sytox™ dead cell dye and mixed with AccuCheck counting beads (both from ThermoFisher Scientific) and a BD FACSymphony™ A1 instrument with an HTS plate loader was used to determine the number of viable cells (Sytox™-negative, proliferated, GFP-positive BCR-expressing cells) per µl by normalizing to the concentration of AccuCheck beads. Cell viability was calculated and plotted as percentage of respective vehicle-treated controls.

BH3-profiling

Mitochondrial BH3-profiling was performed as described earlier.[7,8] Briefly, thawed PBMCs from CLL patients were cultured for 24 h in RPMI-1640 supplemented with 20% FBS, without or with 1 µM ibrutinib, for baseline or dynamic BH3-profiling, respectively. Cells were labeled for 15 min with a PC7-conjugated anti-CD19 antibody (dilution 1:100; cat. No. IM3628, Beckman Coulter), washed, and then stained for 15 min with the fixable viability dye Zombie Violet™ (dilution 1:1000; cat. No. 423113, BioLegend). After washing, the cells were permeabilized with digitonin (0.002%) and exposed for 1 h to different concentrations of BH3-peptides or venetoclax, DMSO as negative control, or 25 µM alamethicin (BML-A150-0005, Enzo) as a positive control for release of cytochrome c. Thereafter, the cells were fixed with paraformaldehyde for 10 min, and after neutralization with tris-glycine buffer they were stained with an AF488-conjugated anti-cytochrome c antibody (dilution 1:3800; cat. No. 612308, BioLegend) overnight at 4° C. Samples were measured on a CytoFLEX XS flow cytometer (Beckman Coulter) and the data were analyzed using FlowJo 10.7.1 (Becton Dickinson). For the dynamic BH3-profiling, delta priming to ibrutinib was calculated as the difference of cytochrome c release between treated and control viable (not stained by Zombie Violet™) $CD19^+$ cells.[9]

Supplemental Tables

Table S1. List of BCRs used for signaling analyses. Provided in a separate Excel file (Supporting Information S2).

Table S2. Baseline (cyto)genetic characteristics of patients in the CLL12 trial.

Cytogenetic and mutational data	Ibrutinib	Placebo	Watch & Wait	Total
**All patients [ITT], N**	**182**	**181**	**152**	**515**
**IGHV mutational status, N (%)**	**181**	**181**	**152**	**514**
Unmutated	70 (38.7)	70 (38.7)	8 (5.3)	148 (28.8)
Mutated	109 (60.2)	109 (60.2)	144 (94.7)	362 (70.4)
Not evaluable	2 (1.1)	2 (1.1)	0 (0)	4 (0.8)
Missing information	1 (0.5)	0 (0)	0 (0)	1 (0.2)
**Cytogenetic subgroup by hierarchical order, N (%)**	**182**	**181**	**152**	**515**
Deletion 17p	6 (3.3)	7 (3.9)	0 (0.0)	13 (2.5)
Deletion 11q	21 (11.5)	19 (10.5)	0 (0.0)	40 (7.8)
Trisomy 12	24 (13.2)	28 (15.5)	4 (2.6)	56 (10.9)
No abnormalities	36 (19.8)	30 (16.6)	27 (17.8)	93 (18.1)
Deletion 13q	95 (52.2)	97 (53.6)	121 (79.6)	313 (60.8)
**Deletion in 17p, N (%)**	**182**	**181**	**152**	**515**
Not present	176 (96.7)	174 (96.1)	152 (100.0)	502 (97.5)
Present	6 (3.3)	7 (3.9)	0 (0)	13 (2.5)
**Deletion in 11q, N (%)**	**182**	**181**	**152**	**515**
Not present	161 (88.5)	162 (89.5)	152 (100.0)	475 (92.2)
Present	21 (11.5)	19 (10.5)	0 (0)	40 (7.8)
**Trisomy 12, N (%)**	**182**	**181**	**152**	**515**
Not present	156 (85.7)	149 (82.3)	148 (97.4)	453 (88.0)
Present	26 (14.3)	32 (17.7)	4 (2.6)	62 (12.0)
**Deletion in 13q, N (%)**	**182**	**181**	**152**	**515**
Not present	61 (33.5)	53 (29.3)	29 (19.1)	143 (27.8)
Present	121 (66.5)	128 (70.7)	123 (80.9)	372 (72.2)
***TP53*, N (%)**	**182**	**181**	**152**	**515**
Unmutated	164 (90.1)	168 (92.8)	148 (97.4)	480 (93.2)
Mutated	18 (9.9)	13 (7.2)	4 (2.6)	35 (6.8)
***NOTCH1*, N (%)**	**182**	**181**	**152**	**515**

Unmutated	167 (91.8)	158 (87.3)	151 (99.3)	476 (92.4)
Mutated	15 (8.2)	23 (12.7)	1 (0.7)	39 (7.6)
***SF3B1*, N (%)**	**182**	**181**	**152**	**515**
Unmutated	168 (92.8)	161 (89.0)	148 (97.4)	477 (92.6)
Mutated	14 (7.7)	20 (11.0)	4 (2.6)	38 (7.4)

Table S3. Comparison of methods for detection of IGLV3-21^{R110}. Provided in a separate Excel file (Supporting Information S2).

Table S4. IGHV genes used and their mutational status. Provided in a separate Excel file (Supporting Information S2).

Table S5. Hazard ratios with 95% confidence intervals and p values for univariate comparisons of EFS between subgroups based on IGLV3-21 rearrangement and IGLV3-21^{G110}/IGLV3-21^{R110}-expression in the whole CLL12 trial.

Cox regression EFS		Hazard ratio	95% confidence interval		P value
			Lower bound	Upper bound	
IGLV3-21^{G110}	***vs.*** Non-IGLV3-21	2.159	0.884	5.275	0.091
IGLV3-21^{R110}	***vs.*** Non-IGLV3-21	3.556	2.148	5.890	<0.001
Non-IGLV3-21	***vs.*** IGLV3-21^{G110}	0.463	0.190	1.131	0.091
IGLV3-21^{R110}	***vs.*** IGLV3-21^{G110}	1.647	0.607	4.466	0.327
Non-IGLV3-21	***vs.*** IGLV3-21^{R110}	0.281	0.170	0.466	<0.001
Unmutated IGHV & IGLV3-21^{R110}	***vs.*** IGLV3-21^{R110}	0.607	0.224	1.647	0.327

Table S6. Hazard ratios with 95% confidence intervals and p values for univariate comparisons of OS between subgroups based on IGLV3-21 rearrangement and IGLV3-21^{G110}/IGLV3-21^{R110}-expression in the whole CLL12 trial.

Cox regression OS		Hazard ratio	95% confidence interval		P value
			Lower bound	Upper bound	
IGLV3-21^{G110}	***vs.*** Non-IGLV3-21	NE	NE	NE	NE
IGLV3-21^{R110}	***vs.*** Non-IGLV3-21	1.277	0.305	5.347	0.737
Non-IGLV3-21	***vs.*** IGLV3-21^{G110}	NE	NE	NE	NE
IGLV3-21^{R110}	***vs.*** IGLV3-21^{G110}	NE	NE	NE	NE
Non-IGLV3-21	***vs.*** IGLV3-21^{R110}	0.783	0.187	3.277	0.737
Unmutated IGHV & IGLV3-21^{R110}	***vs.*** IGLV3-21^{R110}	NE	NE	NE	NE

NE, not evaluable.

Table S7. Hazard ratios with 95% confidence intervals and p values for univariate comparisons of EFS between subgroups based on IGLV3-21 rearrangement and IGLV3-21^{G110}/IGLV3-21^{R110}-expression in the watch & wait arm of the CLL12 trial.

Cox regression EFS		Hazard ratio	95% confidence interval		P value
			Lower bound	Upper bound	
IGLV3-21^{G110}	***vs.*** Non-IGLV3-21	NE	NE	NE	NE
IGLV3-21^{R110}	***vs.*** Non-IGLV3-21	16.807	4.923	57.381	<0.001
Non-IGLV3-21	***vs.*** IGLV3-21^{G110}	NE	NE	NE	NE
IGLV3-21^{R110}	***vs.*** IGLV3-21^{G110}	NE	NE	NE	NE
Non-IGLV3-21	***vs.*** IGLV3-21^{R110}	0.059	0.017	0.203	<0.001
Unmutated IGHV & IGLV3-21^{R110}	***vs.*** IGLV3-21^{R110}	NE	NE	NE	NE

NE, not evaluable.

Table S8. Hazard ratios with 95% confidence intervals and p values for univariate comparisons of OS between subgroups based on IGLV3-21 rearrangement and IGLV3-21^{G110}/IGLV3-21^{R110}-expression in the watch & wait arm of the CLL12 trial.

Cox regression OS		Hazard ratio	95% confidence interval		P value
			Lower bound	Upper bound	
IGLV3-21^{G110}	***vs.*** Non-IGLV3-21	NE	NE	NE	NE
IGLV3-21^{R110}	***vs.*** Non-IGLV3-21	30.424	2.736	338.255	0.005
Non-IGLV3-21	***vs.*** IGLV3-21^{G110}	NE	NE	NE	NE
IGLV3-21^{R110}	***vs.*** IGLV3-21^{G110}	NE	NE	NE	NE
Non-IGLV3-21	***vs.*** IGLV3-21^{R110}	0.033	0.003	0.365	0.005
Unmutated IGHV & IGLV3-21^{R110}	***vs.*** IGLV3-21^{R110}	NE	NE	NE	NE

NE, not evaluable.

Table S9. Hazard ratios with 95% confidence intervals and p values for univariate comparisons of EFS between subgroups based on IGHV mutational status and IGLV3-21^{R110}-expression in the whole CLL12 trial.

Cox regression EFS		Hazard ratio	95% confidence interval		P value
			Lower bound	Upper bound	
Unmutated IGHV & IGLV3-21^{R110}	***vs.*** Unmutated IGHV & no IGLV3-21^{R110}	1.141	0.280	4.651	0.854
Mutated IGHV & no IGLV3-21^{R110}	***vs.*** Unmutated IGHV & no IGLV3-21^{R110}	0.314	0.227	0.433	<0.001
Mutated IGHV & IGLV3-21^{R110}	***vs.*** Unmutated IGHV & no IGLV3-21^{R110}	1.584	0.879	2.855	0.126
Unmutated IGHV & no IGLV3-21^{R110}	***vs.*** Unmutated IGHV & IGLV3-21^{R110}	0.876	0.215	3.572	0.854
Mutated IGHV & no IGLV3-21^{R110}	***vs.*** Unmutated IGHV & IGLV3-21^{R110}	0.275	0.067	1.123	0.072
Mutated IGHV & IGLV3-21^{R110}	***vs.*** Unmutated IGHV & IGLV3-21^{R110}	1.388	0.313	6.160	0.666
Unmutated IGHV & no IGLV3-21^{R110}	***vs.*** Mutated IGHV & no IGLV3-21^{R110}	3.186	2.309	4.396	<0.001
Unmutated IGHV & IGLV3-21^{R110}	***vs.*** Mutated IGHV & no IGLV3-21^{R110}	3.635	0.890	14.842	0.072
Mutated IGHV & IGLV3-21^{R110}	***vs.*** Mutated IGHV & no IGLV3-21^{R110}	5.047	2.793	9.118	<0.001
Unmutated IGHV & no IGLV3-21^{R110}	***vs.*** Mutated IGHV & IGLV3-21^{R110}	0.631	0.350	1.138	0.126
Unmutated IGHV & IGLV3-21^{R110}	***vs.*** Mutated IGHV & IGLV3-21^{R110}	0.720	0.162	3.197	0.666
Mutated IGHV & no IGLV3-21^{R110}	***vs.*** Mutated IGHV & IGLV3-21^{R110}	0.198	0.110	0.358	<0.001

Table S10. Hazard ratios with 95% confidence intervals and p values for univariate comparisons of EFS between subgroups based on IGHV mutational status and IGLV3-21^{R110}-expression in the ibrutinib arm of the CLL12 trial.

Cox regression EFS		Hazard ratio	95% confidence interval		P value
			Lower bound	Upper bound	
Unmutated IGHV & IGLV3-21^{R110}	***vs.*** Unmutated IGHV & no IGLV3-21^{R110}	1.606	0.214	12.031	0.645
Mutated IGHV & no IGLV3-21^{R110}	***vs.*** Unmutated IGHV & no IGLV3-21^{R110}	0.572	0.291	1.127	0.106
Mutated IGHV & IGLV3-21^{R110}	***vs.*** Unmutated IGHV & no IGLV3-21^{R110}	2.443	0.830	7.195	0.105
Unmutated IGHV & no IGLV3-21^{R110}	***vs.*** Unmutated IGHV & IGLV3-21^{R110}	0.623	0.083	4.666	0.645
Mutated IGHV & no IGLV3-21^{R110}	***vs.*** Unmutated IGHV & IGLV3-21^{R110}	0.356	0.047	2.706	0.319
Mutated IGHV & IGLV3-21^{R110}	***vs.*** Unmutated IGHV & IGLV3-21^{R110}	1.521	0.169	13.657	0.708
Unmutated IGHV & no IGLV3-21^{R110}	***vs.*** Mutated IGHV & no IGLV3-21^{R110}	1.747	0.888	3.439	0.106
Unmutated IGHV & IGLV3-21^{R110}	***vs.*** Mutated IGHV & no IGLV3-21^{R110}	2.806	0.370	21.298	0.319
Mutated IGHV & IGLV3-21^{R110}	***vs.*** Mutated IGHV & no IGLV3-21^{R110}	4.268	1.415	12.879	0.010
Unmutated IGHV & no IGLV3-21^{R110}	***vs.*** Mutated IGHV & IGLV3-21^{R110}	0.409	0.139	1.205	0.105
Unmutated IGHV & IGLV3-21^{R110}	***vs.*** Mutated IGHV & IGLV3-21^{R110}	0.657	0.073	5.900	0.708
Mutated IGHV & no IGLV3-21^{R110}	***vs.*** Mutated IGHV & IGLV3-21^{R110}	0.234	0.078	0.707	0.010

Table S11. Hazard ratios with 95% confidence intervals and p values for univariate comparisons of OS between subgroups based on IGHV mutational status and IGLV3-21^{R110}-expression in the whole CLL12 trial.

Cox regression OS		Hazard ratio	95% confidence interval		P value
			Lower bound	Upper bound	
Unmutated IGHV & IGLV3-21^{R110}	***vs.*** Unmutated IGHV & no IGLV3-21^{R110}	3.718	0.477	28.956	0.21
Mutated IGHV & no IGLV3-21^{R110}	***vs.*** Unmutated IGHV & no IGLV3-21^{R110}	0.744	0.354	1.564	0.436
Mutated IGHV & IGLV3-21^{R110}	***vs.*** Unmutated IGHV & no IGLV3-21^{R110}	0.706	0.091	5.468	0.739
Unmutated IGHV & no IGLV3-21^{R110}	***vs.*** Unmutated IGHV & IGLV3-21^{R110}	0.269	0.035	2.095	0.21
Mutated IGHV & no IGLV3-21^{R110}	***vs.*** Unmutated IGHV & IGLV3-21^{R110}	0.2	0.027	1.503	0.118
Mutated IGHV & IGLV3-21^{R110}	***vs.*** Unmutated IGHV & IGLV3-21^{R110}	0.19	0.012	3.047	0.241
Unmutated IGHV & no IGLV3-21^{R110}	***vs.*** Mutated IGHV & no IGLV3-21^{R110}	1.344	0.639	2.824	0.436
Unmutated IGHV & IGLV3-21^{R110}	***vs.*** Mutated IGHV & no IGLV3-21^{R110}	4.995	0.665	37.515	0.118
Mutated IGHV & IGLV3-21^{R110}	***vs.*** Mutated IGHV & no IGLV3-21^{R110}	0.948	0.127	7.086	0.959
Unmutated IGHV & no IGLV3-21^{R110}	***vs.*** Mutated IGHV & IGLV3-21^{R110}	1.417	0.183	10.976	0.739
Unmutated IGHV & IGLV3-21^{R110}	***vs.*** Mutated IGHV & IGLV3-21^{R110}	5.267	0.328	84.524	0.241
Mutated IGHV & no IGLV3-21^{R110}	***vs.*** Mutated IGHV & IGLV3-21^{R110}	1.054	0.141	7.877	0.959

Table S12. Hazard ratios with 95% confidence intervals and p values for univariate comparisons of OS between subgroups based on IGHV mutational status and IGLV3-21^{R110}-expression in the ibrutinib arm of the CLL12 trial.

Cox regression OS		Hazard ratio	95% confidence interval		P value
			Lower bound	Upper bound	
Unmutated IGHV & IGLV3-21^{R110}	***vs.*** Unmutated IGHV & no IGLV3-21^{R110}	7.196	0.795	65.116	0.079
Mutated IGHV & no IGLV3-21^{R110}	***vs.*** Unmutated IGHV & no IGLV3-21^{R110}	1.313	0.384	4.49	0.664
Mutated IGHV & IGLV3-21^{R110}	***vs.*** Unmutated IGHV & no IGLV3-21^{R110}	NE	NE	NE	NE
Unmutated IGHV & no IGLV3-21^{R110}	***vs.*** Unmutated IGHV & IGLV3-21^{R110}	0.139	0.015	1.258	0.079
Mutated IGHV & no IGLV3-21^{R110}	***vs.*** Unmutated IGHV & IGLV3-21^{R110}	0.183	0.022	1.498	0.113
Mutated IGHV & IGLV3-21^{R110}	***vs.*** Unmutated IGHV & IGLV3-21^{R110}	NE	NE	NE	NE
Unmutated IGHV & no IGLV3-21^{R110}	***vs.*** Mutated IGHV & no IGLV3-21^{R110}	0.761	0.223	2.603	0.664
Unmutated IGHV & IGLV3-21^{R110}	***vs.*** Mutated IGHV & no IGLV3-21^{R110}	5.479	0.668	44.966	0.113
Mutated IGHV & IGLV3-21^{R110}	***vs.*** Mutated IGHV & no IGLV3-21^{R110}	NE	NE	NE	NE
Unmutated IGHV & no IGLV3-21^{R110}	***vs.*** Mutated IGHV & IGLV3-21^{R110}	NE	NE	NE	NE
Unmutated IGHV & IGLV3-21^{R110}	***vs.*** Mutated IGHV & IGLV3-21^{R110}	NE	NE	NE	NE
Mutated IGHV & no IGLV3-21^{R110}	***vs.*** Mutated IGHV & IGLV3-21^{R110}	NE	NE	NE	NE

NE, not evaluable.

Table S13. Univariable analysis of candidates for the multivariable modelling.

COX regression EFS		Hazard ratio	95% Confidence Interval		P value
			Lower bound	Upper bound	
**Treatment arm**					
Ibrutinib	***vs.*** Placebo	0.321	0.221	0.468	<0.001
**Age (years)**					
> 60	***vs.*** ≤ 60	1.147	0.801	1.641	0.454
**Gender**					
Male	***vs.*** Female	0.966	0.656	1.422	0.862
**B symptoms**					
Yes	***vs.*** No	1.365	0.434	4.290	0.594
**ECOG performance status score**					
≥ 1	***vs.*** 0	1.277	0.785	2.076	0.324
**CIRS score**					
> 1	***vs.*** ≤ 1	1.278	0.868	1.881	0.214
**Creatinine clearance (Cockroft-Gault) (ml/min)**					
≥ 70	***vs.*** < 70	1.164	0.743	1.826	0.507
**Serum β2-microglobulin (mg/L)**					
> 3.5	***vs.*** ≤ 3.5	1.959	1.159	3.313	0.012
**Serum thymidine kinase (U/L)**					
> 10	***vs.*** ≤ 10	1.574	0.995	2.488	0.052
**IGHV mutational status**					
Unmutated	***vs.*** Mutated	2.589	1.826	3.673	<0.001
**Deletion in 17p**					
Yes	***vs.*** No	2.691	1.362	5.315	0.004
**Deletion in 11q**					
Yes	***vs.*** No	1.832	1.159	2.897	0.010
**Trisomy in 12**					
Yes	***vs.*** No	1.728	1.123	2.657	0.013
**Deletion in 13q**					
Yes	***vs.*** No	0.809	0.563	1.162	0.251
TP53					
Mutated	***vs.*** Unmutated	1.546	0.902	2.651	0.113
**IGLV3-21**					
Usage	***vs.*** No usage	2.514	1.543	4.095	<0.001
**IGLV3-21^{R110}**					
Yes	***vs.*** No	2.479	1.423	4.318	0.001
**Subset2**					
Yes	***vs.*** No	1.344	0.497	3.636	0.561

Table S14. Correlation of IGLV3-21^{R110} expression with the GCLLSG score and with its constituent risk factors.

Patient characteristics	No IGLV3-21^{R110} expression	IGLV3-21^{R110} expression	Total	P value
**All patients [ITT], N**	**490**	**25**	**515**	
**GCLLSG score at baseline, N (%)**	**487**	**23**	**510**	**Mann- whitney U-test**
Mean [SD]	3.7 [2.1]	4.3 [1.3]	3.8 [2.0]	0.063
Median	4	4	4	
Interquartile range	2-5	4-5	2-5	
Range	0-12	2-6	0-12	
**Age (categorical), N (%)**	**490**	**25**	**515**	**Exact Fisher test**
≤ 60	226 (46.1)	10 (40.0)	236 (45.8)	0.682
> 60	264 (53.9)	15 (60.0)	279 (54.2)	
**Sex, N (%)**	**490**	**25**	**515**	**Exact Fisher test**
Female	177 (36.1)	7 (28.0)	184 (35.7)	0.523
Male	313 (63.9)	18 (72.0)	331 (64.3)	
**β2-microglobulin (categorical), N (%)**	**490**	**25**	**515**	**Chi² test**
≤ 1.7 mg/L	95 (19.4)	0 (0)	95 (18.4)	0.049
> 1.7 mg/L & ≤ 3.5 mg/L	369 (75.3)	23 (92.0)	392 (76.1)	
> 3.5 mg/L	26 (5.3)	2 (8.0)	28 (5.4)	
**ECOG performance status (categorical), N (%)**	**490**	**25**	**515**	**Exact Fisher test**
0	449 (91.6)	23 (92.0)	472 (91.7)	1.000
> 0	41 (8.4)	2 (8.0)	43 (8.3)	
**Serum thymidine kinase (categorical), N (%)**	**490**	**25**	**515**	**Exact Fisher test**
≤ 10 U/L	212 (43.3)	5 (20.0)	217 (42.1)	0.023
> 10 U/L	278 (56.7)	20 (80.0)	298 (57.9)	
**IGHV mutational status, N (%)**	**487**	**23**	**510**	**Exact Fisher test**
Unmutated	143 (29.4)	5 (21.7)	148 (29.0)	0.492
Mutated	344 (70.6)	18 (78.3)	362 (71.0)	
Missing information	3 (0.6)	2 (8.0)	5 (1.0)	
**Deletion in 17p, N (%)**	**490**	**25**	**515**	**Exact Fisher test**
No	477 (97.3)	25 (100.0)	502 (97.5)	1.000
Yes	13 (2.7)	0 (0)	13 (2.5)	
Missing information	0 (0)	0 (0)	0 (0)	
**Deletion in 11q, N (%)**	**490**	**25**	**515**	**Exact Fisher test**
No	453 (92.4)	22 (88.0)	475 (92.2)	0.432
Yes	37 (7.6)	3 (12.0)	40 (7.8)	
Missing information	0 (0)	0 (0)	0 (0)	

Supplemental Figures

#92757 (IGLV3-21^{G110})

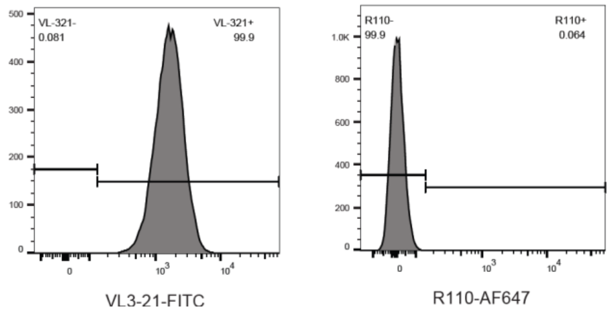

#23845 (IGLV3-21^{G110})

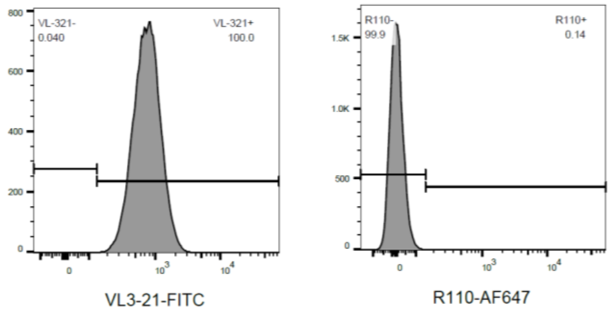

#3062 (IGLV3-21^{R110})

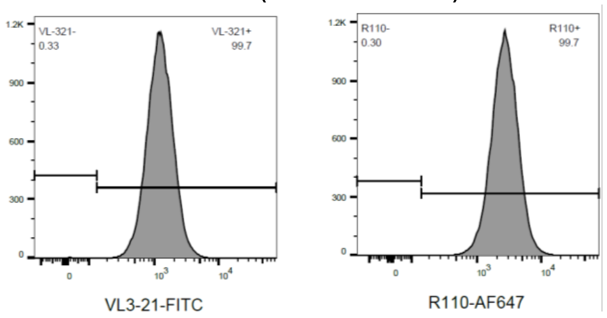

#21704 (IGLV3-21^{R110})

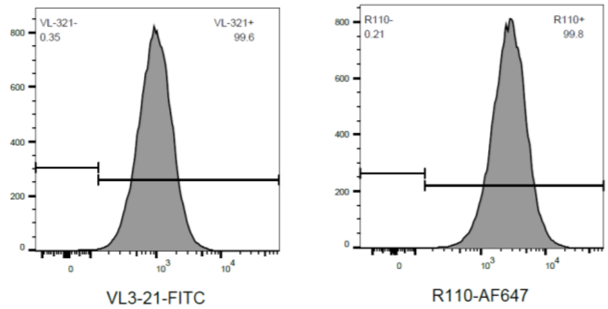

#97462 (IGLV3-21^{R110})

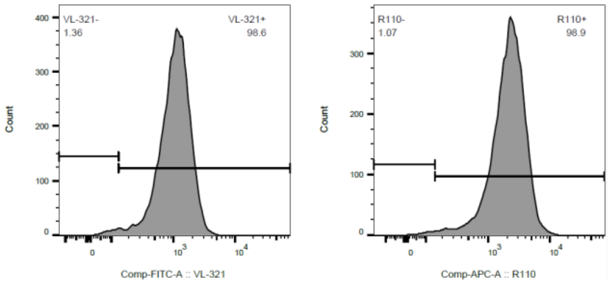

#97915 (IGLV3-21^{R110})

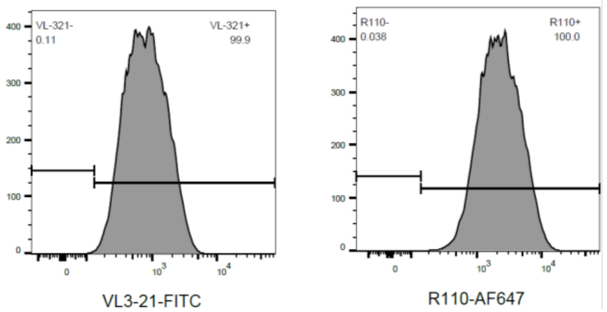

#49962 (IGLV3-21^{G110})

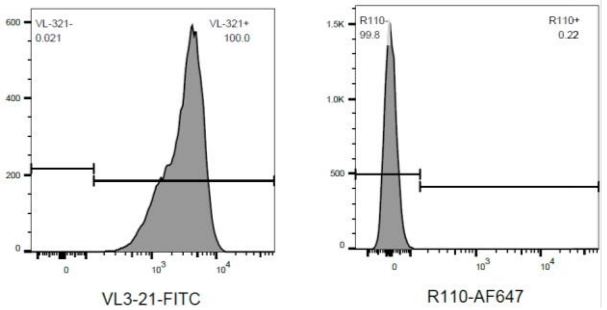

#51975 (IGLV3-21^{R110})

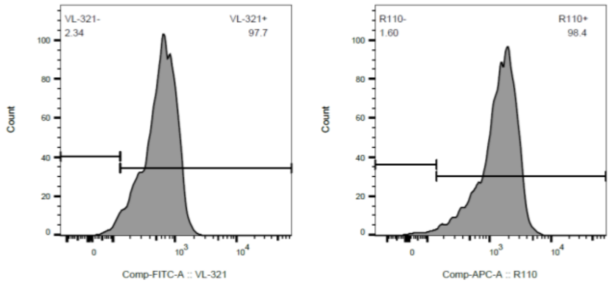

#31251 (no IGLV3-21)

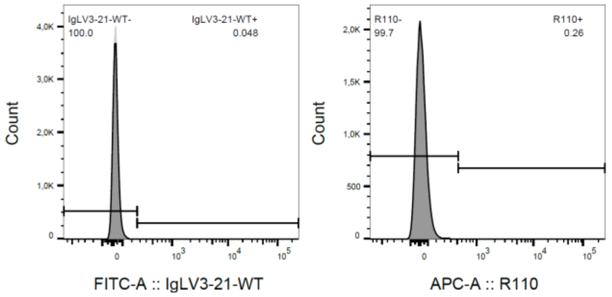

#45439 (no IGLV3-21)

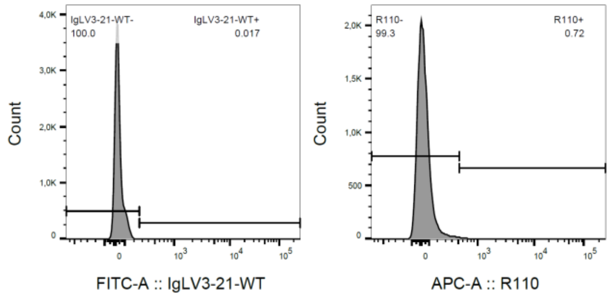

continues on the next page…

#66601 (no IGLV3-21)

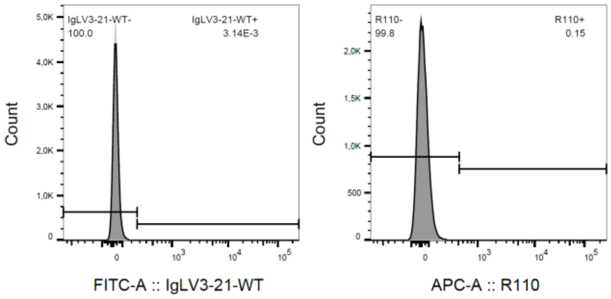

#70986 (IGLV3-21^{R110})

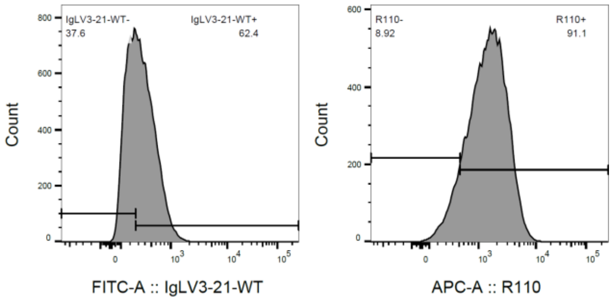

#94476 (no IGLV3-21)

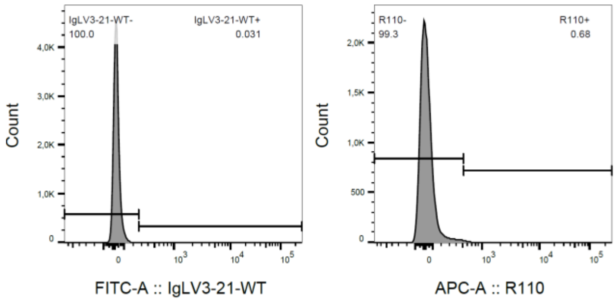

#59951 (no IGLV3-21)

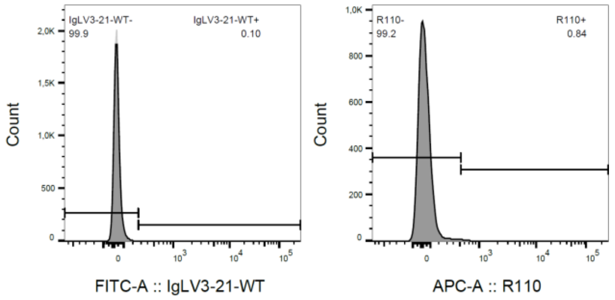

#12081 (no IGLV3-21)

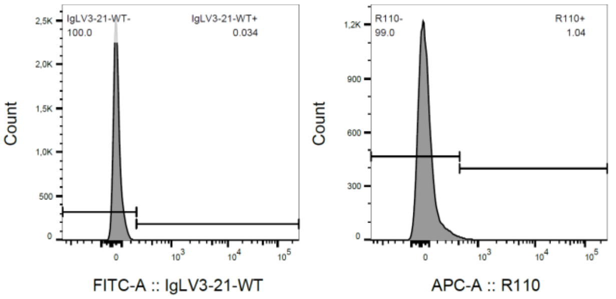

#12178 (no IGLV3-21)

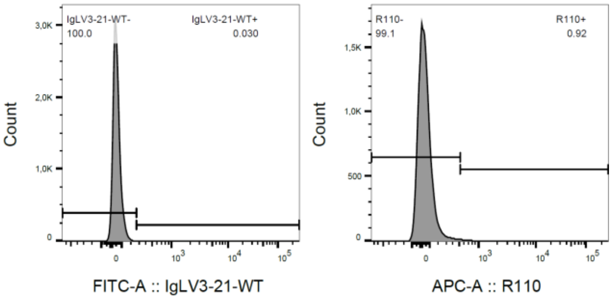

#36371 (IGLV3-21^{R110})

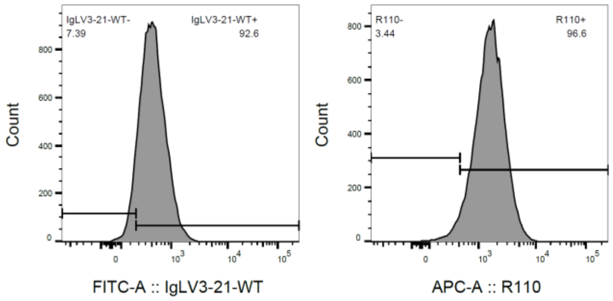

#99977 (no IGLV3-21)

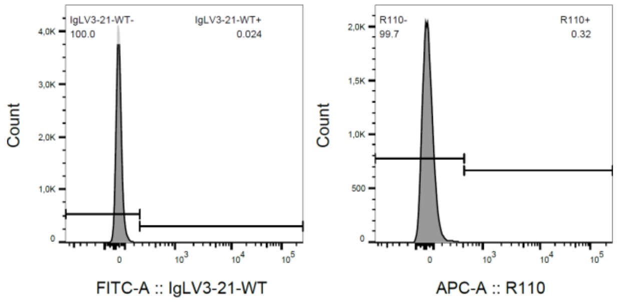

#57916 (no IGLV3-21)

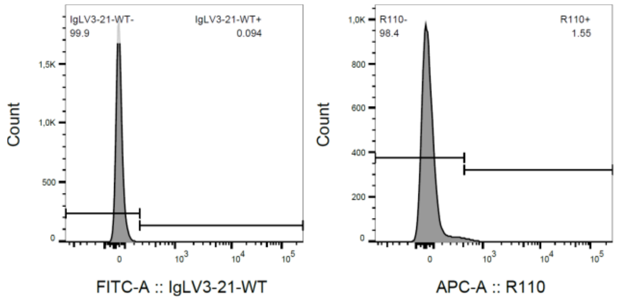

#6607 (no IGLV3-21)

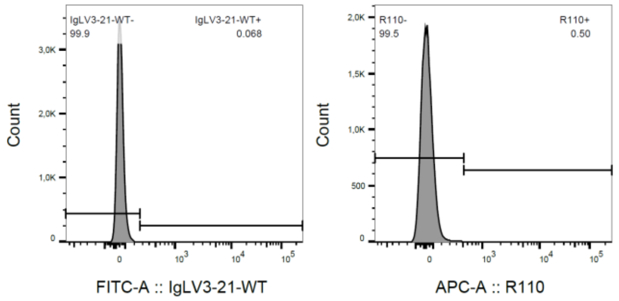

continues on the next page…

#59159 (IGLV3-21^{R110})

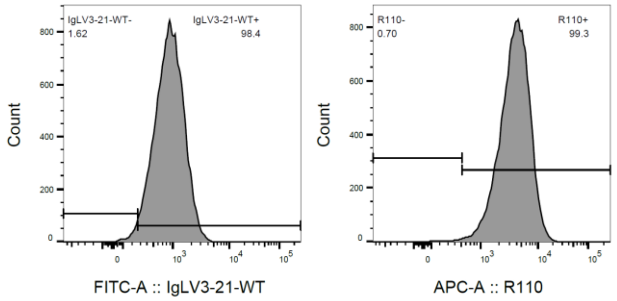

#61191 (IGLV3-21^{R110})

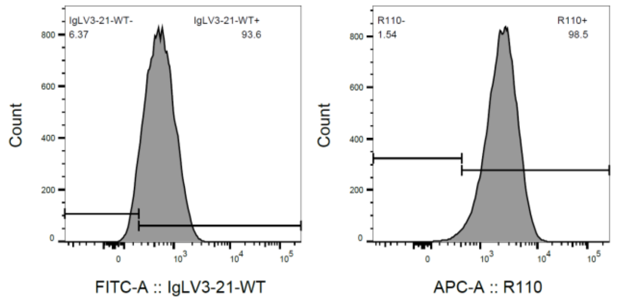

#89669 (IGLV3-21^{R110})

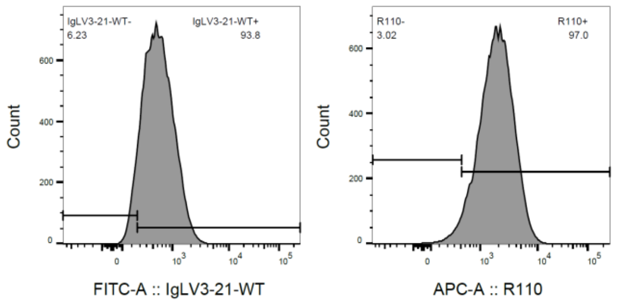

#73489 (IGLV3-21^{G110})

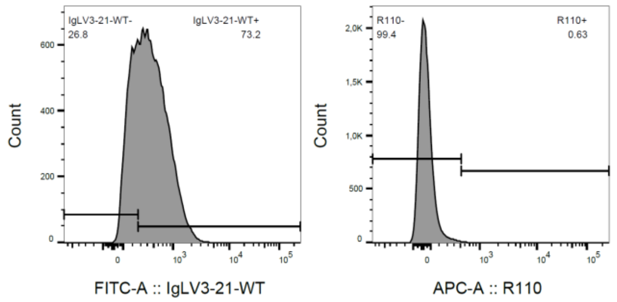

#23082 (IGLV3-21^{R110})

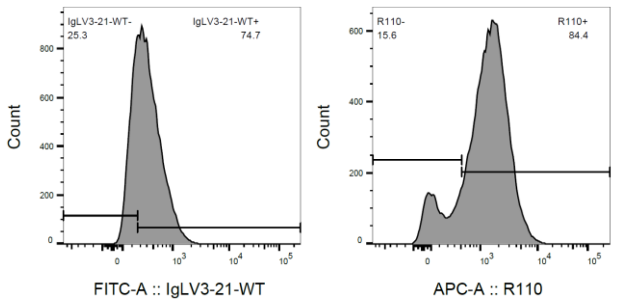

#20088 (IGLV3-21^{R110})

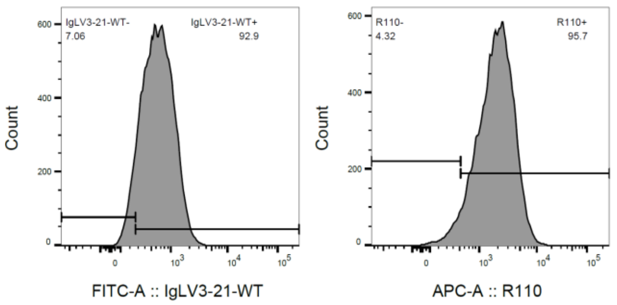

#17902 (IGLV3-21^{R110})

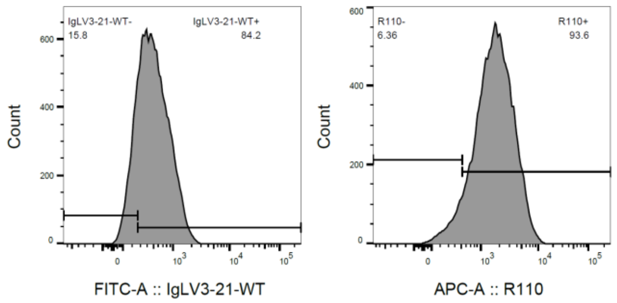

#27909 (IGLV3-21^{R110})

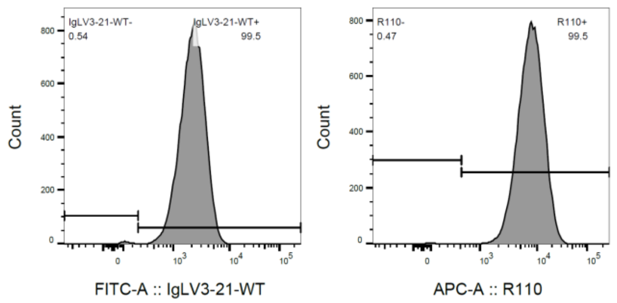

#71798 (IGLV3-21^{G110})

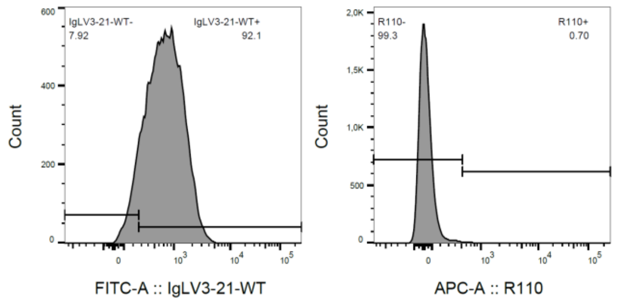

#85621 (IGLV3-21^{G110})

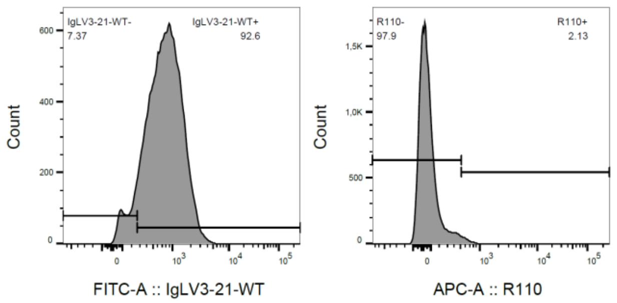

continues on the next page…

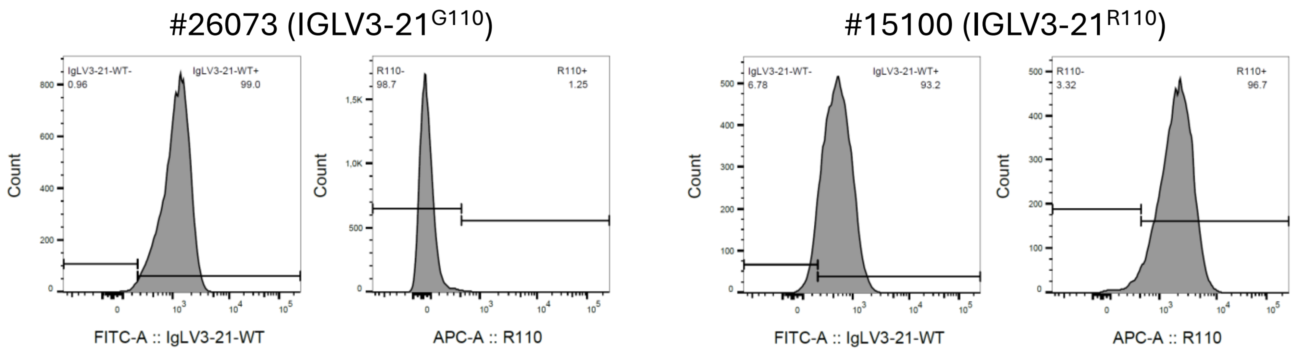

Figure S1. Immunophenotyping using specific antibodies against IGLV3-21^{G110} and IGLV3-21^{R110}. Samples from 32 CLL patients were subjected to flow cytometry and initially gated to select the CD19^{+}/CD5^{+} cell population. Left and right panels for each sample show histograms for expression of IGLV3-21^{G110} (wild-type) and IGLV3-21^{R110} (mutated), respectively. Numbers denote the percentage of cells in the respective gate.

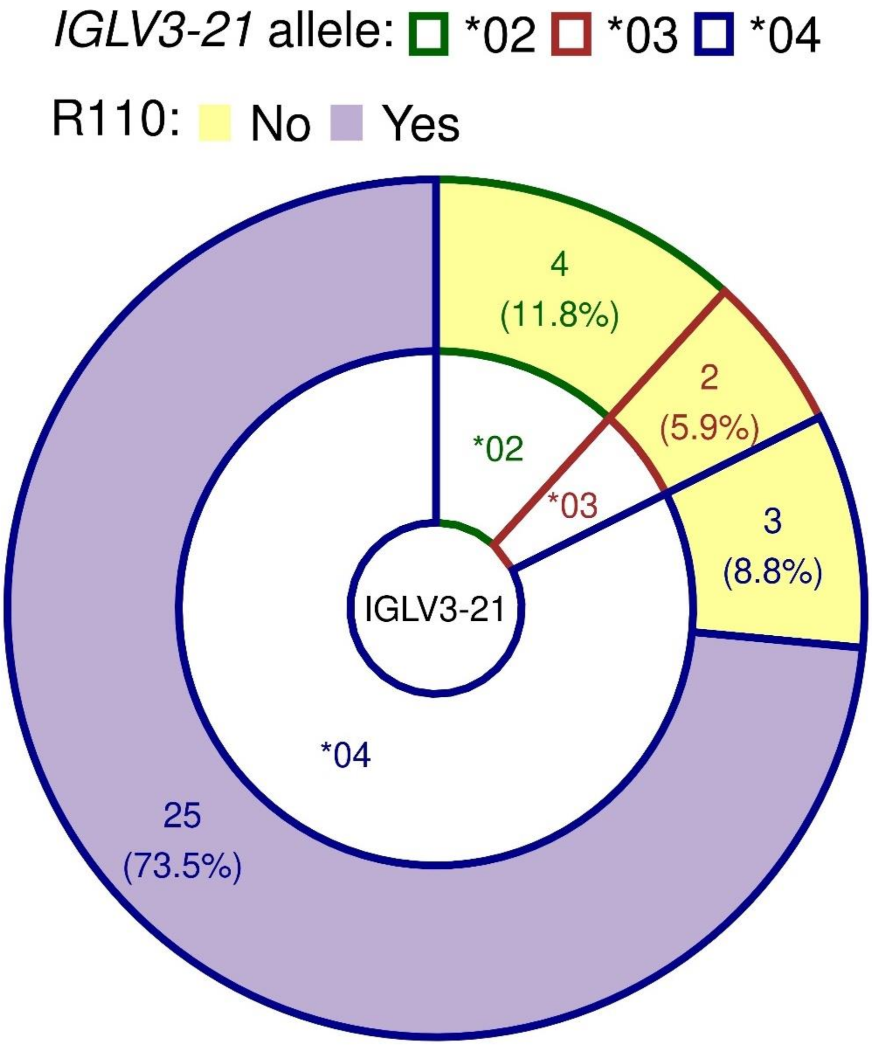

Figure S2. ***IGLV3-21*** **allele and presence or absence of G110R mutation among the cases expressing IGLV3-21.**

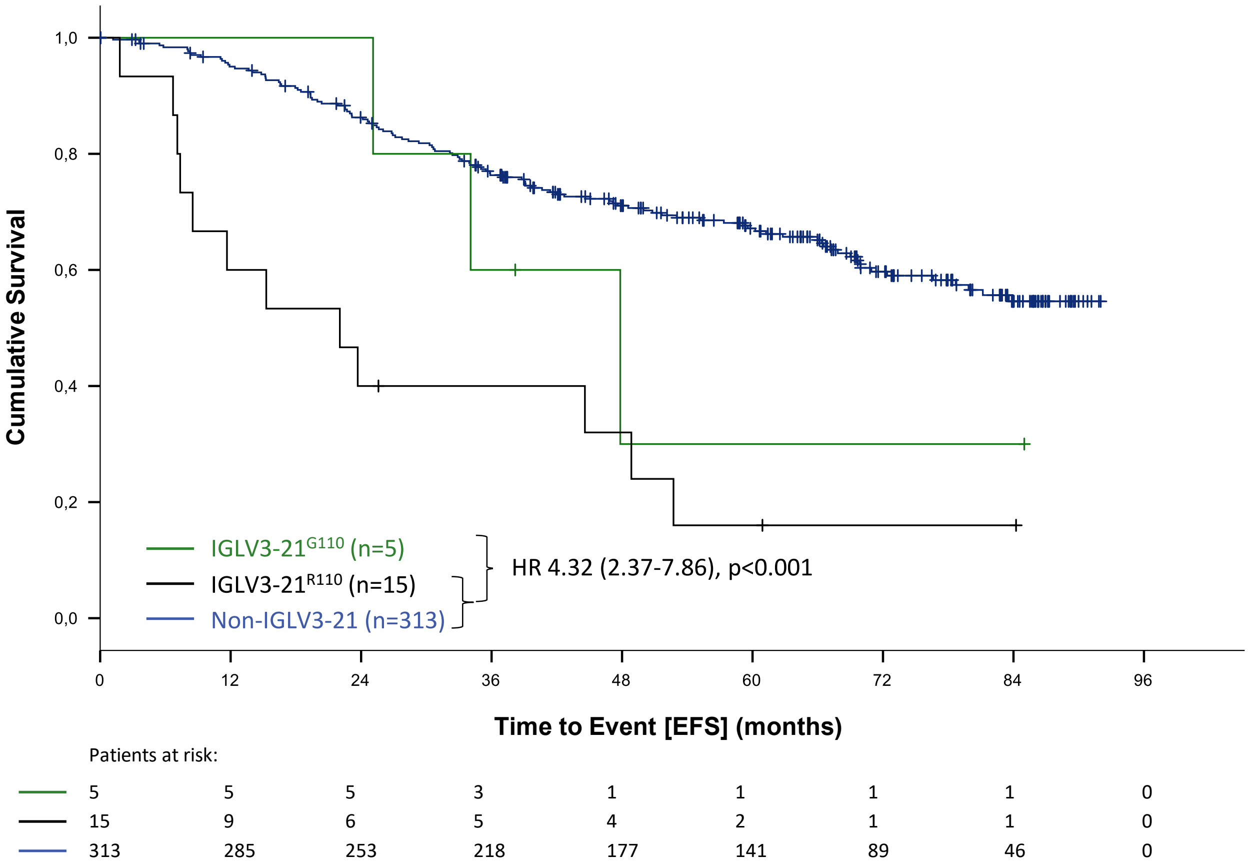

Cox regression EFS		Hazard ratio	95% confidence interval		P value
			Lower bound	Upper bound	
IGLV3-21^{G110}	***vs.*** Non-IGLV3-21	1.85	0.59	5.82	0.295
IGLV3-21^{R110}	***vs.*** Non-IGLV3-21	4.37	2.4	7.97	< 0.001
IGLV3-21^{R110}	***vs.*** IGLV3-21^{G110}	2.37	0.67	8.41	0.182

Figure S3. Kaplan-Meier plot and risk table estimating EFS of patients from the watch & wait and placebo cohorts of the CLL12 trial pooled together. Hazard ratios (HR) with 95% confidence interval and p values were calculated using the Cox proportional hazards regression model with Wald test.

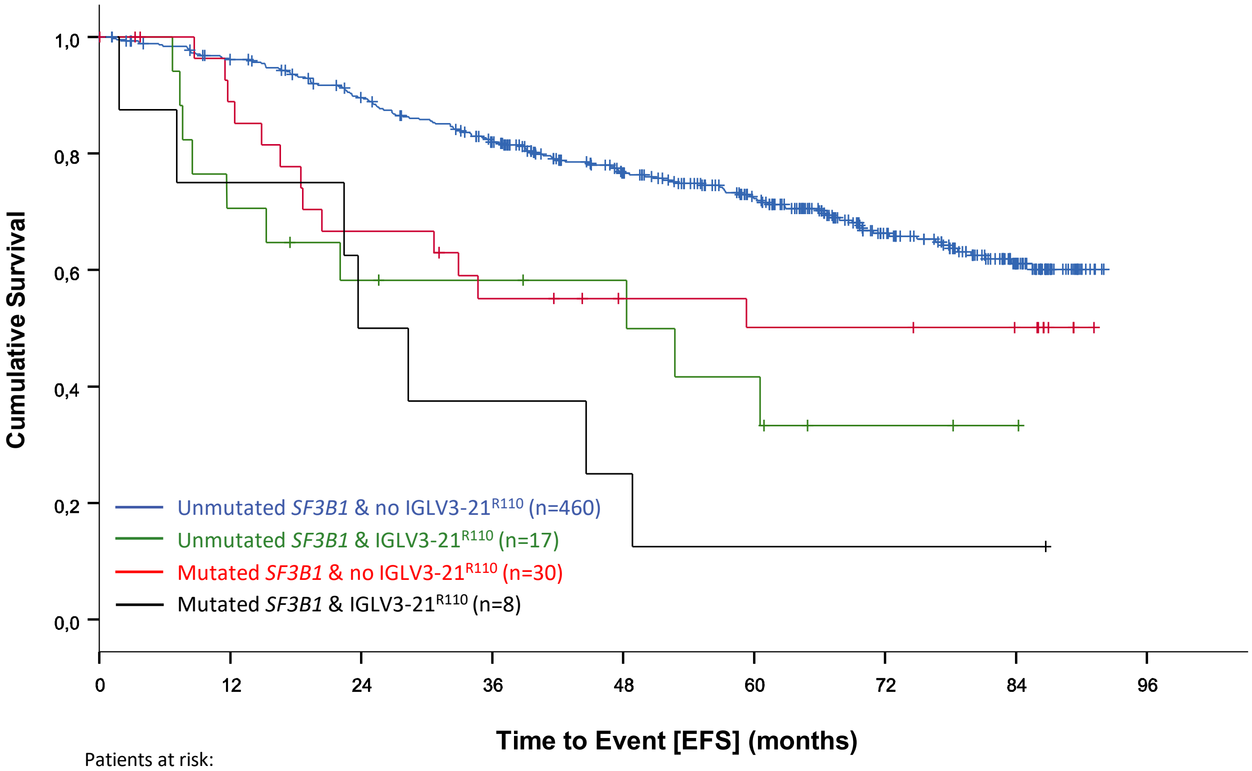

Patients at risk:

Unmutated *SF3B1* & no IGLV3-21R110 (blue)	460	417	379	335	269	216	139	73	0
Unmutated *SF3B1* & IGLV3-21R110 (green)	17	12	9	8	7	5	2	1	0
Mutated *SF3B1* & no IGLV3-21R110 (red)	30	24	18	14	11	10	10	8	0
Mutated *SF3B1* & IGLV3-21R110 (black)	8	6	4	3	2	1	1	1	0

COX regression EFS		Hazard ratio	95% confidence interval		P value
			Lower bound	Upper bound	
Unmutated *SF3B1* & IGLV3-21^{R110}	***vs.*** Unmutated *SF3B1* & no IGLV3-21^{R110}	3.071	1.614	5.845	< 0.001
Mutated *SF3B1* & IGLV3-21^{R110}	***vs.*** Mutated *SF3B1* & no IGLV3-21^{R110}	2.704	1.077	6.789	0.034
Mutated *SF3B1* & no IGLV3-21^{R110}	***vs.*** Unmutated *SF3B1* & no IGLV3-21^{R110}	1.815	1.026	3.209	0.04
Mutated *SF3B1* & IGLV3-21^{R110}	***vs.*** Unmutated *SF3B1* & IGLV3-21^{R110}	1.598	0.607	4.201	0.342
Unmutated *SF3B1* & IGLV3-21^{R110}	***vs.*** Mutated *SF3B1* & no IGLV3-21^{R110}	1.693	0.741	3.868	0.212
Mutated *SF3B1* & IGLV3-21^{R110}	***vs.*** Unmutated *SF3B1* & no IGLV3-21^{R110}	4.907	2.291	10.51	< 0.001

Figure S4. EFS of patients from all trial arms stratified according to presence or absence of *SF3B1* mutations and presence of IGLV3-21^{R110}. Hazard ratios, 95% confidence intervals, and p values were calculated using the Cox proportional hazards regression model with Wald test.

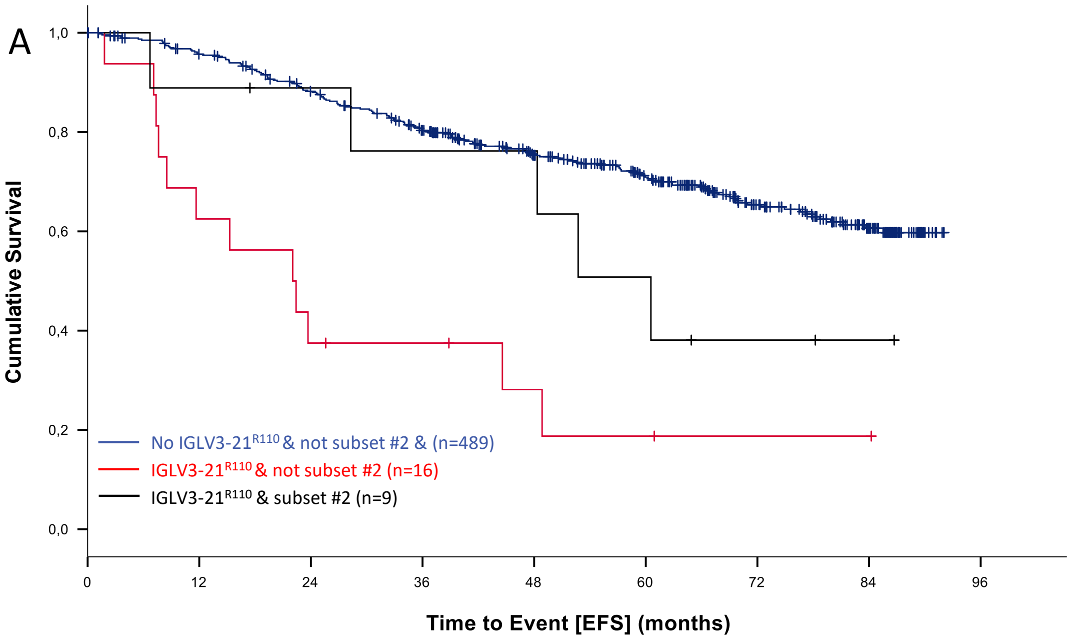

Patients at risk:

No IGLV3-21^{R110} & not subset #2 (blue)	489	440	396	348	280	226	149	81	0
IGLV3-21^{R110} & not subset #2 (red)	16	10	6	5	3	2	1	1	0
IGLV3-21^{R110} & subset #2 (black)	9	8	7	6	6	4	2	1	0

COX regression EFS		Hazard ratio	95% confidence interval		P value
			Lower bound	Upper bound	
IGLV3-21^{R110} & no subset #2	***vs.*** IGLV3-21^{R110} & subset #2	2.616	0.92	7.441	0.071
IGLV3-21^{R110} & no subset #2	***vs.*** No IGLV3-21^{R110} & no subset #2	5.148	2.846	9.312	<0.001
IGLV3-21^{R110} & subset #2	***vs.*** No IGLV3-21^{R110} & no subset #2	1.968	0.807	4.799	0.137

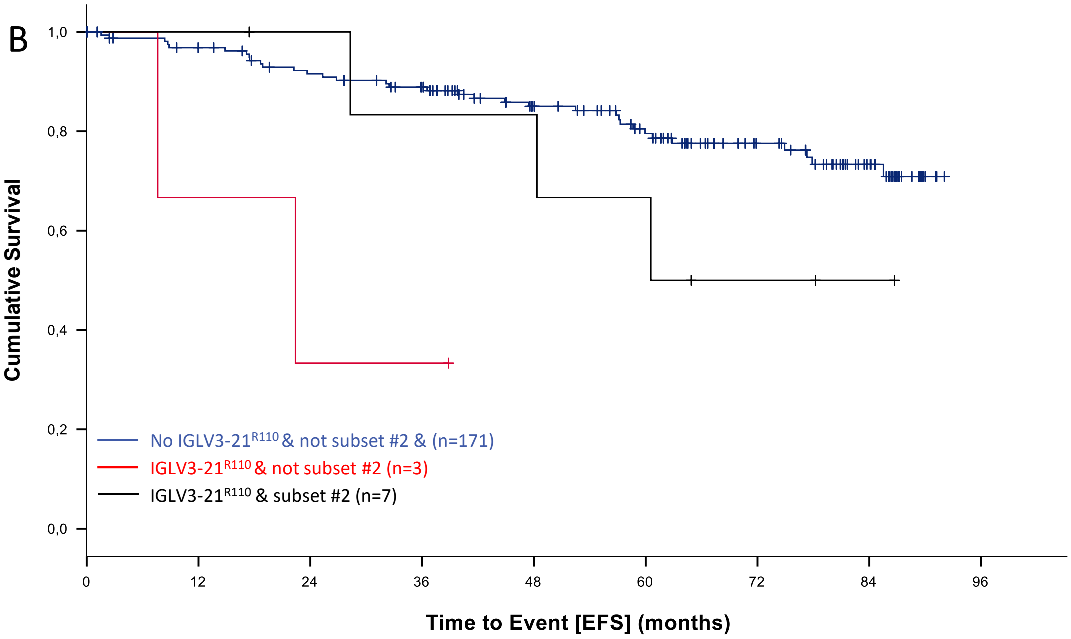

Patients at risk:

No IGLV3-21^{R110} & not subset #2 (blue)	171	150	138	127	102	84	59	34	0
IGLV3-21^{R110} & not subset #2 (red)	3	2	1	1	0	0	0	0	0
IGLV3-21^{R110} & subset #2 (black)	7	7	6	5	5	4	2	1	0

COX regression EFS		Hazard ratio	95% confidence interval		P value
			Lower bound	Upper bound	
IGLV3-21^{R110} & no subset #2	***vs.*** IGLV3-21^{R110} & subset #2	4.561	0.738	28.169	0.102
IGLV3-21^{R110} & no subset #2	***vs.*** No IGLV3-21^{R110} & no subset #2	9.371	2.162	40.62	0.003
IGLV3-21^{R110} & subset #2	***vs.*** No IGLV3-21^{R110} & no subset #2	2.055	0.631	6.692	0.232

Figure S5. EFS of patients from CLL12 stratified according to presence or absence of IGLV3-21^{R110} and belonging or not to stereotyped subset #2. (A) In the whole trial. (B) Only in the ibrutinib arm of the trial.

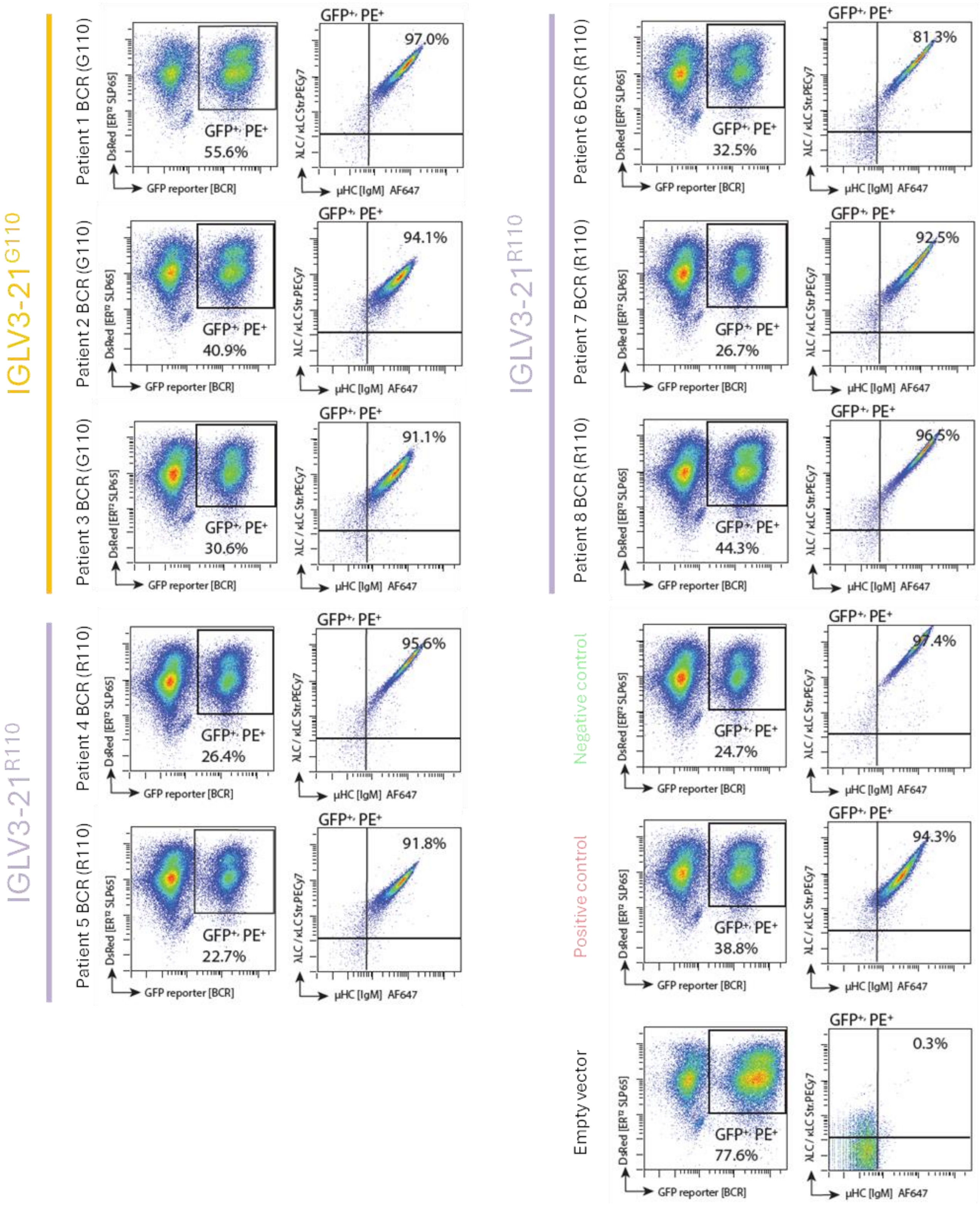

Figure S6. Flow cytometric analysis of successfully transduced TKO-EST cells (GFP-positive, left panels of each column) after surface staining with anti-μ and anti-κ/λ antibodies (right panels of each column). The expressed BCRs include 3 BCRs with IGLV3-21^{G110} LCs, 5 BCRs with IGLV3-21^{R110} LCs, one BCR derived from a healthy donor (negative control) and one strongly reactive BCR from a CLL subset #201 case (positive control). Cells transduced with an empty vector are also shown.

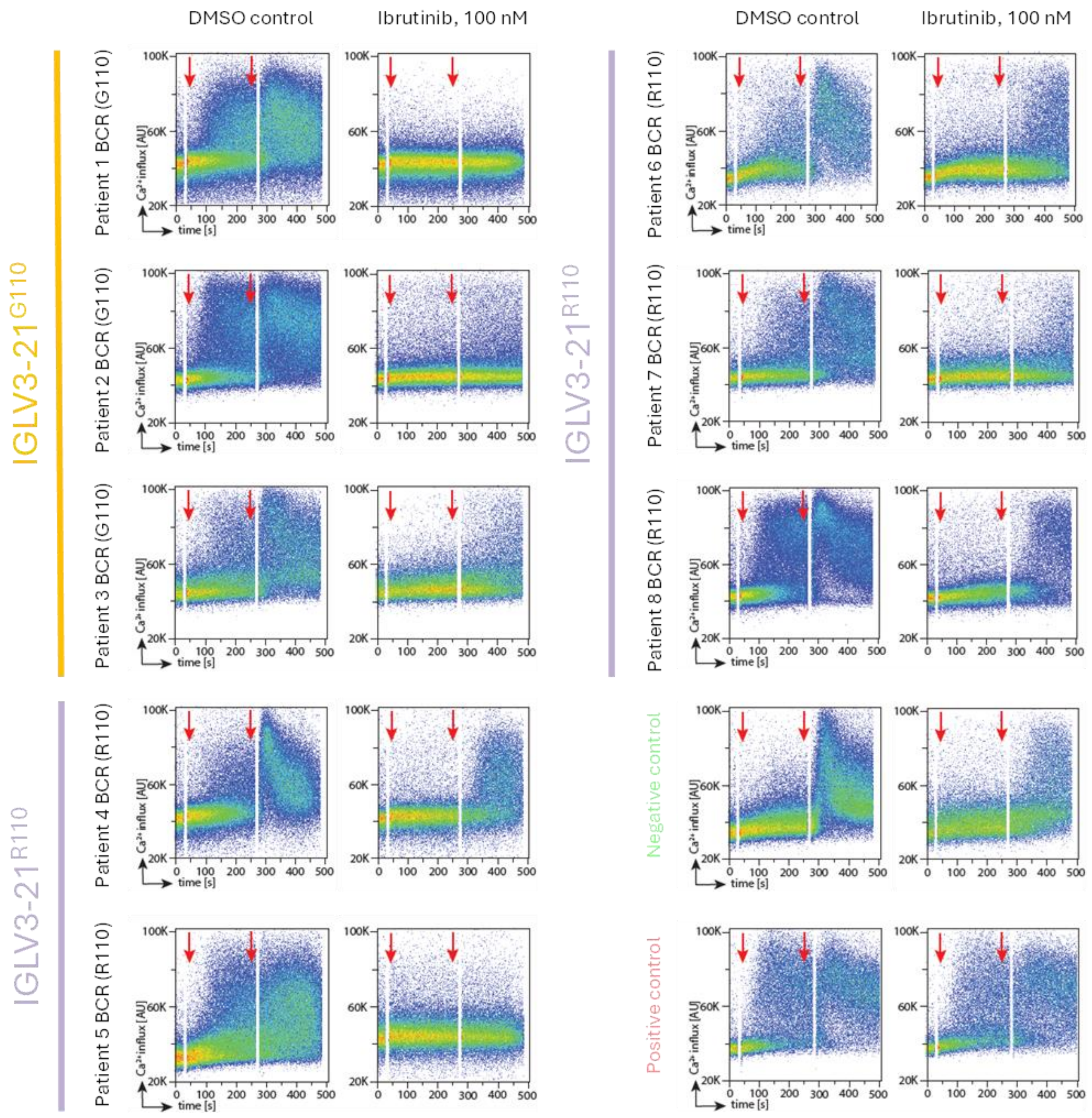

Figure S7. Ca^{2+} influx in TKO-EST cells expressing BCRs from different patients. An autonomously active BCR from a previously analyzed subset #201 CLL patient was used as positive control. A BCR isolated from a naïve B cell from a healthy donor (IGHV4-39/IGKV3-15) was used as negative control for autonomous signaling. On each density plot, the first arrow denotes the addition of 4-hydroxytamoxifen and the second arrow the addition of anti-κ/λ antibodies. One representative measurement is shown for each sample but at least 3 measurements per sample were recorded (summarized in Figures S6 and S7).

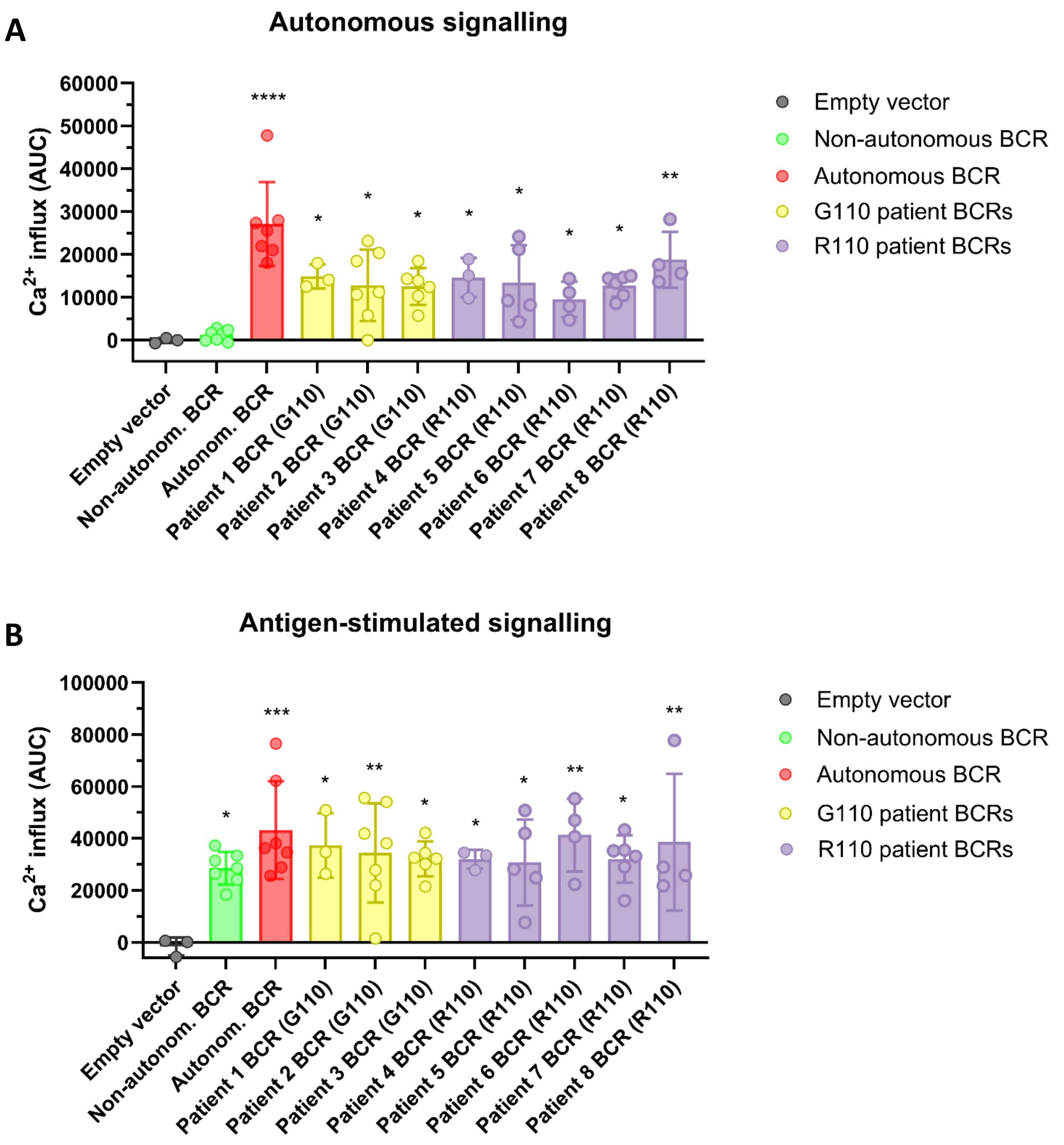

Figure S8. Ca^{2+} influx in TKO-EST cells expressing patient-derived BCRs. (A) Autonomous signaling after treatment of the cells with 4-hydroxytamoxifen that induces the ERT2-SLP65 fusion protein. (B) Antigen-stimulated signaling after treatment with both 4-hydroxitamoxifen and anti-κ/λ antibodies. Three of the BCRs were derived from patient CLL cells expressing IGLV3-21^{G110} and 5 are from patient CLL cells expressing IGLV3-21^{R110}. An autonomously active BCR from a previously analyzed subset #201 CLL patient was used as a positive control. A BCR isolated from a naïve B cell from a healthy donor (IGHV4-39/IGKV3-15) was used as a negative control for autonomous signaling. Statistical significance was evaluated using one-way ANOVA with Dunnett's multiple comparisons test, where all columns were compared to the empty-vector control. *, $P < 0.05$; **, $P < 0.01$; ***, $P < 0.001$; ****, $P < 0.0001$.

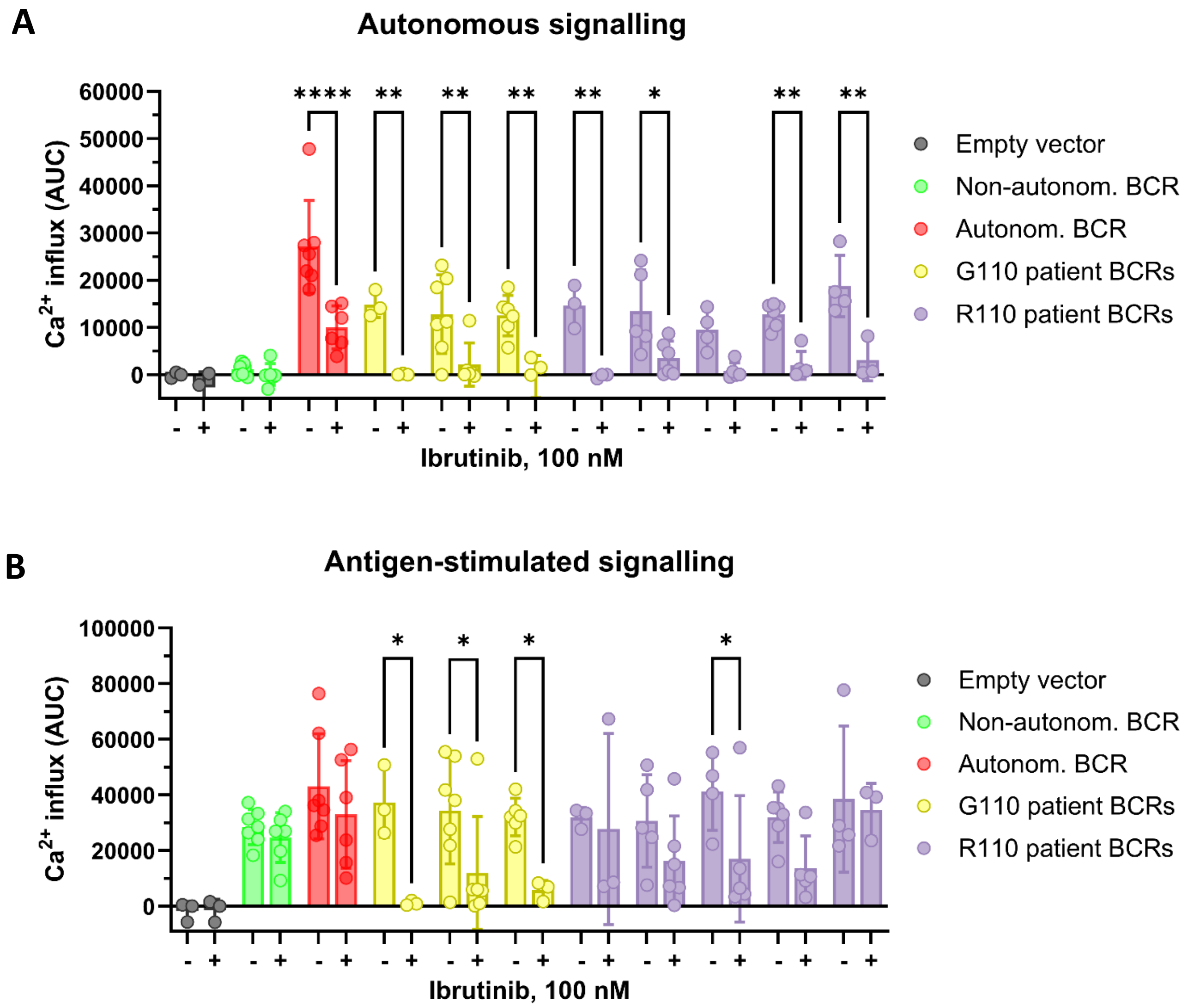

Figure S9. Inhibition of Ca^{2+} influx by ibrutinib in TKO-EST cells expressing patient-derived BCRs and treated with 4-hydroxitamoxifen to induce the ERT2-SLP65 fusion protein. (A) Inhibition of autonomous signaling. (B) Inhibition of antigen-stimulated signaling triggered by treatment with anti-κ/λ antibodies. Three of the BCRs were derived from patient CLL cells expressing IGLV3-21^{G110} and 5 are from patient CLL cells expressing IGLV3-21^{R110}. An autonomously active BCR from a previously analyzed subset #201 CLL patient was used as positive control. A BCR isolated from a naïve B cell from a healthy donor (IGHV4-39/IGKV3-15) was used as negative control for autonomous signaling. Statistical evaluation was performed using a mixed-effects model (akin to 2-way ANOVA) with donor BCR and ibrutinib treatment as factors and Šidák's multiple comparisons test, where every treated column was compared to the corresponding non-treated control. *, $P < 0.05$; **, $P < 0.01$; ****, $P < 0.0001$.

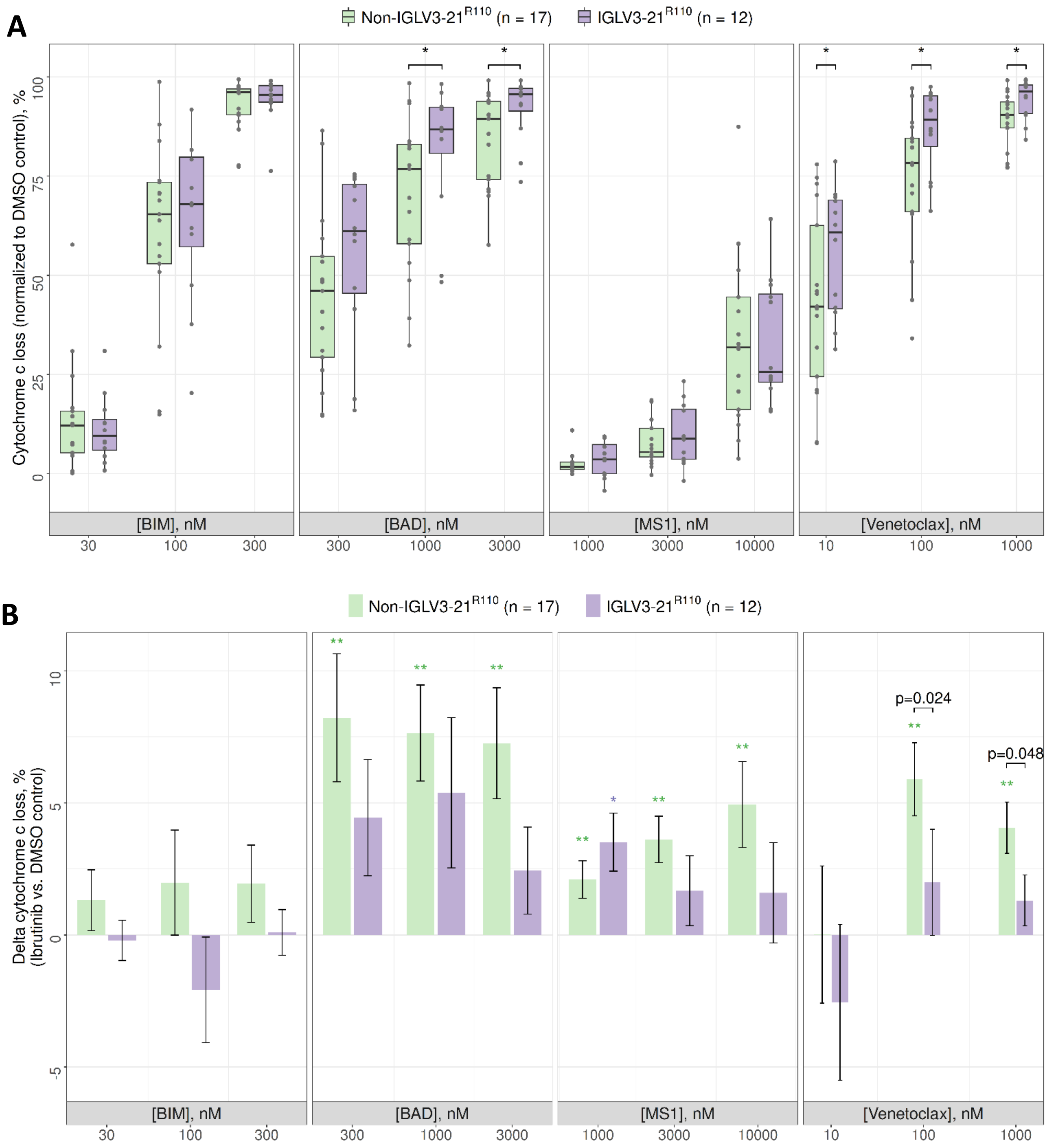

Figure S10. Baseline and dynamic BH3-profiling of primary patient CLL cells expressing IGLV3-21^{R110} (n=12) or not (n=17). (A) Estimation of baseline priming by measurement of the percentage of cells that have lost mitochondrial cytochrome c after incubation with BH3 peptides (BIM, BAD or MS1) or venetoclax for 1 h. Data point on the box plot represent individual patient samples measured in triplicate experiments and averaged. (B) Delta priming of cells from the same patients as in (A) incubated with 1 µM ibrutinib for 24 h before measurement of cytochrome c release as above. The height of the bars indicates the difference in the percentage of cells with cytochrome c loss between the treatment and control condition. Error bars depict the standard error of the mean. Asterisks in (A) denote significant difference (two-sided unpaired Student's t-test with Benjamini-Hochberg adjustment) between patient groups. Asterisks over individual columns in (B) depict statistical significance (two-sided paired Student's t-test with Benjamini-Hochberg adjustment) for the comparison with the vehicle (DMSO) control: *, $q < 0.05$; **, $q < 0.01$. Intergroup comparisons in (B) were evaluated with the Mann-Whitney U test and significant p-values (< 0.05) are displayed.

Supplemental References

1. Krober A, Seiler T, Benner A, et al. V(H) mutation status, CD38 expression level, genomic aberrations, and survival in chronic lymphocytic leukemia. *Blood*. 2002;100(4):1410-1416.
2. Marks JD, Hoogenboom HR, Bonnert TP, McCafferty J, Griffiths AD, Winter G. By-passing immunization. Human antibodies from V-gene libraries displayed on phage. *J Mol Biol*. 1991;222(3):581-597.
3. Bystry V, Agathangelidis A, Bikos V, et al. ARResT/AssignSubsets: a novel application for robust subclassification of chronic lymphocytic leukemia based on B cell receptor IG stereotypy. *Bioinformatics*. 2015;31(23):3844-3846.
4. Brochet X, Lefranc MP, Giudicelli V. IMGT/V-QUEST: the highly customized and integrated system for IG and TR standardized V-J and V-D-J sequence analysis. *Nucleic Acids Res*. 2008;36(Web Server issue):W503-508.
5. Maity PC, Bilal M, Koning MT, et al. IGLV3-21*01 is an inherited risk factor for CLL through the acquisition of a single-point mutation enabling autonomous BCR signaling. *Proc Natl Acad Sci U S A*. 2020;117(8):4320-4327.
6. Nicolo A, Linder AT, Jumaa H, Maity PC. The Determinants of B Cell Receptor Signaling as Prototype Molecular Biomarkers of Leukemia. *Front Oncol*. 2021;11:771669.
7. Jebaraj BMC, Muller A, Dheenadayalan RP, et al. Evaluation of vecabrutinib as a model for noncovalent BTK/ITK inhibition for treatment of chronic lymphocytic leukemia. *Blood*. 2022;139(6):859-875.
8. Montero J, Sarosiek KA, DeAngelo JD, et al. Drug-induced death signaling strategy rapidly predicts cancer response to chemotherapy. *Cell*. 2015;160(5):977-989.
9. Pan RA, Wang Y, Qiu S, et al. BH3 profiling as pharmacodynamic biomarker for the activity of BH3 mimetics. *Haematologica*. 2024;109(4):1253-1258.
